# Insect herbivores should follow plants escaping their relatives

**DOI:** 10.1007/s00442-014-3026-3

**Published:** 2014-07-23

**Authors:** Benjamin Yguel, Richard Ian Bailey, Claire Villemant, Amaury Brault, Hervé Jactel, Andreas Prinzing

**Affiliations:** 1 University of Rennes 1/Centre National de la Recherche Scientifique, Research Unit UMR 6553, « Ecosystems, Biodiversity, Evolution», Campus Beaulieu, Bâtiment 14 A, 263 Av. du Général Leclerc, 35042 Rennes Cedex, France; 2 Centre for Ecological and Evolutionary Synthesis, Department of Biology, University of Oslo, Blindern, P.O. Box 1066, 0316 Oslo, Norway; 3 Muséum National d’Histoire Naturelle, UMR 7205, MNHN-CNRS, CP50, Entomologie, 45 rue Buffon, 75005 Paris, France; 4 Laboratory of Forest Entomology and Biodiversity, UMR BIOGECO-INRA, 69, route d’Arcachon, 33612 Cestas Cedex, France; 5 Alterra Centre for Ecosystem Studies, Wageningen University and Research Centre, PO Box 47, 6700 AA Wageningen, The Netherlands; 6 German Centre for Integrative Biodiversity Research (iDiv) Halle-Jena-Leipzig, Deutscher Platz 5e, 04103 Leipzig, Germany

**Keywords:** Community phylogeny, Macroevolution, Trophic chain, Parasitism rate, Temperate forest

## Abstract

Neighboring plants within a local community may be separated by many millions of years of evolutionary history, potentially reducing enemy pressure by insect herbivores. However, it is not known how the evolutionary isolation of a plant affects the fitness of an insect herbivore living on such a plant, especially the herbivore’s enemy pressure. Here, we suggest that evolutionary isolation of host plants may operate similarly as spatial isolation and reduce the enemy pressure per insect herbivore. We investigated the effect of the phylogenetic isolation of host trees on the pressure exerted by specialist and generalist enemies (parasitoids and birds) on ectophagous Lepidoptera and galling Hymenoptera. We found that the phylogenetic isolation of host trees decreases pressure by specialist enemies on these insect herbivores. In Lepidoptera, decreasing enemy pressure resulted from the density dependence of enemy attack, a mechanism often observed in herbivores. In contrast, in galling Hymenoptera, enemy pressure declined with the phylogenetic isolation of host trees per se, as well as with the parallel decline in leaf damage by non-galling insects. Our results suggest that plants that leave their phylogenetic ancestral neighborhood can trigger, partly through simple density-dependency, an enemy release and fitness increase of the few insect herbivores that succeed in tracking these plants.

**Electronic supplementary material:**

The online version of this article (doi:10.1007/s00442-014-3026-3) contains supplementary material, which is available to authorized users.

## Introduction

Plant communities differ drastically in phylogenetic proximity of their incumbent species. Neighboring plants may belong to the same or a closely related species, or a species may be evolutionarily separated by dozens of millions of years. Because of widespread phylogenetic conservatism in host plant use by insect herbivores (e.g., Futuyma and Agrawal 2009), phylogenetically diverse plant communities may have higher arthropod diversity (Castagneyrol and Jactel 2012). It has also been demonstrated that a given plant facing distantly related neighbors may suffer less enemy pressure than a plant facing closely related neighbors (Yguel et al. 2011; see also Gilbert and Webb 2007; Violle et al. 2011), thus potentially increasing the fitness of plants escaping closely related plant neighbors (e.g., Rousset and Lepart 2000). However, it remains unknown how the evolutionary isolation of a plant from its local neighbors affects the fitness of an insect herbivore living on such a phylogenetically isolated plant. The fitness of insect herbivores can be strongly dependent on the level of enemy pressure, often causing a high number of mortalities (Hairston et al. 1960). It remains unclear to what extent the release of a phylogenetically isolated plant from its herbivore enemies translates into a release of each of the few remaining herbivores living on such a plant from their own enemies.

It is well-established that the isolation of host plants in space can release herbivores from their enemies (Kruess and Tscharntke 1994; Holt et al. 1999), albeit the opposite has also been observed; for instance, if enemies are more efficient than herbivores in finding plant hosts or if they can attack herbivores feeding on multiple plant hosts (Bruckmann et al. 2011). The spatial isolation of plant hosts can hence increase the fitness of herbivores (Kondoh 2003). The phylogenetic isolation of host plants might operate similarly to spatial isolation, as phylogenetically distant plant neighbors tend to share only a few insect herbivores (Vialatte et al. 2010; Bertheau et al. 2010), resulting in a decline in herbivory that is distinctly stronger than the decline due to spatial isolation (Yguel et al. 2011). The phylogenetic isolation of a host plant could hence also affect the enemy pressure suffered by an herbivore attacking such a plant. Phylogenetic isolation of the host plant might negatively affect enemy pressure on an insect herbivore because enemies often use olfactory cues emitted by host plants to locate their prey (Mills 1993; Dicke and Van Loon 2000; Rott et al. 2005; Mantyla et al. 2008), leading to the added difficulty of finding both the prey’s host plant and the prey itself. Moreover, the decline in herbivore abundances may trigger reduced attack rates by natural enemies, which often decline with prey rarefaction (Crawley 1983). In such a case, an insect herbivore on a phylogenetically isolated plant would benefit from enemy release. However, enemy pressure could also increase with the phylogenetic isolation of host plants if the natural enemies are less constrained by the phylogenetic isolation of plant hosts than the herbivores themselves—for instance, due to more effective host-seeking behavior, greater dispersal capacity, less specialized resource use, or lower intra-guild predation pressure. Overall, enemy pressure on an insect herbivore may depend on host-plant phylogenetic isolation, but the form of this relationship would depend on both the diet breadth and the foraging capacities of natural enemies compared to those of herbivores.

Using a continuous gradient of phylogenetic isolation of oaks from their neighboring trees, we studied whether oak tree phylogenetic isolation decreases or increases the enemy pressure of specialist (parasitoids) and generalist (birds) enemies of oak insect herbivores (ectophagous Lepidoptera). In addition, we analyzed *how* the phylogenetic isolation of tree hosts affects the enemy pressure of herbivores: directly or, rather, indirectly, i.e., transmitted via other direct causes. As such other direct causes we considered (a) the density of insect herbivores and the related herbivory, as both may attract enemies (Crawley 1983) and decline with host-tree phylogenetic isolation (Yguel et al. 2011), and (b) tree budburst phenology, which can strongly influence insect herbivore density and associated herbivory, and hence, the parasitism rate (Wesolowski and Rowinski 2008; Le Corff et al. 2000; Rott et al. 2005).We compared these effects of phylogenetic isolation of host trees to two other well-known drivers of insect herbivore density and rates of parasitism or predation: the diversity of surrounding trees (e.g., Koricheva et al. 2006) and oak spatial isolation (Ozanne et al. 2000). We add similar analyses of a biologically very different group of phytophages, i.e., Hymenopteran galls, and their chalcidoid parasitoids in the Appendix (in Electronic Supplementary Material).

## Methods

### Site description and experimental design

Our study was conducted in 2010 and 2011, in the State Forest of Rennes (area 2,000 ha), in Brittany (France; see details in Appendix S1). We studied adult oaks. as the adult cohort represents by far the largest volume of hosts for insect herbivores, and each single adult oak represents a separate resource patch for both insect herbivores and their enemies, much more so than a seedling or juvenile oak. Crowns of oaks surrounded by pines or others gymnosperms were always located below the dominant canopy. Thus, we also chose to sample among the phylogenetically less isolated oaks when they had crowns below the dominant canopy. All sampled trees had similar heights. The mean tree height estimated from pictures and cross multiplications was 10.68 ± 2.76 m. Given that this study is on adult trees, experimentation is virtually impossible and we took a correlative approach, profiting from a natural experiment. Twenty-two ca. 60-year-old oaks were chosen for the study, with age estimated according to tree size (mean circumference ± SD at breast height 62.1 ± 16.7 cm) and information from local forest managers. We used a pair-wise design, with one oak surrounded mainly by oak and beech, and the other far from any other oak or beech, and mainly surrounded by pines and other gymnosperms of angiosperms distantly related to oaks. A total of 17 tree species were observed in contact with the focal oaks. Trees within a pair were close to each other (30–150 m apart), and belonged to the same oak species, *Quercus petraea* or *Q. robur* (note that these oak species are closely related and can hybridize). Pairs were spread across the entire forest. Such an approach of blocking has been recommended to parcel out spatially varying environmental conditions, as their impacts are relatively constant within a block (Legendre et al. 2004). Neither tree pair nor tree species had any effect on parasitism or on predation rates of insect herbivores; we present the effects of pair or species in Appendix S2.

### Phylogenetic isolation of host trees within the surrounding canopy

For each focal oak, we quantified its mean phylogenetic distance to all neighboring trees with which its crown was in contact. Phylogenetic distances were extracted from published phylogenies (Appendix S3) following procedures applied previously (Vialatte et al. 2010; Yguel et al. 2011), using phylogenetic classification (APG 2009). To quantify phylogenetic distances we used the younger of the crown ages of the two lineages involved on the respective level of classification (crown age is the age of earliest diversification within lineage, contrary to stem age, the age of the common ancestor separating the two tree species). For instance, we ranked the comparison between oak and pine species as a comparison between two classes, gymnosperms and angiosperms, between which the younger is approximately 140 million years old (the crown age of angiosperms), and the phylogenetic distance is hence 140 million years. We opted against stem ages because they would give too much weight to the gymnosperm neighbors, being separated from angiosperms since >300 million years ago. Moreover, crown age is biologically more informative than stem age: it represents the time when the oak lineage and the other lineage started to be physically and physiologically distinct from the point of view of insect herbivores. A table of phylogenetic distances between tree taxa can be found in Appendix S3.

Overall, the phylogenetic isolation of oaks ranged from 10 to 125.66 million years, and varied continuously between these extremes. It has been previously shown that the angiosperm understory is not a major source of colonists for the angiosperm canopy (Gossner et al. 2009), and therefore, understory tree species (<6 m height) were not taken into account for estimating phylogenetic isolation.

### Tree diversity and spatial isolation

Because plant community diversity and spatial isolation are known to affect insect predation pressure (Koricheva et al. 2006), we calculated the species diversity of canopy trees surrounding the focal oaks, the species richness of the surrounding canopy, and a measure of spatial isolation: distance (in m) to the closest oak. Diversity was calculated using 1 minus the Simpson’s diversity index (Rosenzweig 1995). The effects of diversity, species richness, or spatial isolation on parasitism and predation rate are presented in the “Results”. See Appendix S4 for effects on other dependent variables.

### Budburst phenology and the physical and chemical tree environment

Budburst phenology was measured by scoring the phenological state of ten random apical buds from the upper layer of the crown every three days from 15 March to 31 May. The phenological state corresponds to a three-rank scale described in Wesolowski and Rowinski (2008). We calculated the number of days required to reach a score of 20, corresponding to the maximum score for the ten apical buds (day 0 corresponding to the earliest tree with budburst = 20; for more details of the sampling procedure, see Yguel et al. 2011, Appendix S3). Multiple other environmental factors potentially influencing parasitism rate or bird predation were measured (crown size, crown volume, surrounding canopy density, temperature and humidity, leaf C/N ratios, and leaf dry weight), but were uncorrelated with parasitism or predation rates of ectophagous Lepidoptera. The results are presented in Appendix S5.

### Ectophagous Lepidoptera and their natural enemies

We studied the density and parasitism rates of ectophagous lepidopteran larvae and the diversity of their insect parasitoids in spring 2010 and 2011. The spring period corresponds to the peak of ectophagous Lepidoptera larval density on oaks (Southwood et al. 2004). As described in Yguel et al. (2011), larvae were sampled twice each year, in early and late spring. The first sample was collected just after the budburst of all trees, and the second sample was collected three weeks later. Two meters of branches were cut in the upper and lower strata of each tree and larvae were manually collected from all leaves of the cut branches. We sampled 206 larvae from 9,739 leaves in 2010 and 203 from 14,914 leaves in 2011. The density of ectophagous lepidopteran larvae was calculated as the number of larvae divided by the number of leaves sampled. The most abundant species of Lepidoptera were oligophagous (O) or monophagous (M) [in decreasing abundance *Archips* spp. (O), *Tortrix viridana* (M), *Hedya nubiferana* (O), *Orthosia cerasi* (O) and *Conistra erythrocephala* (O) in 2010 and *Archips* sp.(O), *O. cerasi* (O), *Carcina quercana* (O), *T. viridana* (M), *H. nubiferana* (O) in 2011], with oligophagous species showing a distinct preference for, but no restriction to, oaks or Fagaceae (see Yguel et al. 2011 for references). All collected larvae were put in Petri dishes at ambient temperature in the laboratory and fed every two days with fresh oak leaves until pupation. Ectoparasitoids were recorded directly after leaf sampling; endoparasitoids were recorded upon emergence from larvae or pupae for up to five months after leaf sampling. Fifty-one larvae were parasitized in 2010 and 67 in 2011. The parasitism rate was calculated as the ratio of the total number of larvae parasitized by endo- and ectoparasitoids to the total number of larvae collected on each tree. In 2010, one tree had 100 % parasitism based on a sample with only one larva. This observation was considered as an extreme outlier and was therefore excluded from all analyses.

Ninety-four endoparasitoid specimens were identified to the species or morphospecies level. The identification of species is given in Appendix S6. Endoparasitoid species richness was not correlated with phylogenetic isolation of the focal oaks nor with density of the ectophagous Lepidoptera larvae when endoparasitoid density was included as a co-variable (Appendix S7).

We note that the parasitism rate might be somewhat underestimated in our study. First, some parasitized larvae may not have been recorded as such because larvae or pupae died for other reasons before parasitoids could emerge. However, this error should not bias our results, as rearing conditions were identical for larvae from all trees. Second, some larval parasitism can also occur after spring. However, spring is the peak period of larval density on oaks (Southwood et al. 2004), and hence it is the decisive time window for their control by parasitoids.

We also explored possible a numerical bias: densities of ectophagous Lepidoptera larvae might correlate to parasitism rates simply because parasitism rate is calculated as the number of parasitized larvae divided by larval density. We attributed random larval densities to our 22 trees, constrained between 0 and18. This range of larval densities corresponds to the observed ranges from 0 to 22 (in 2010) or 18 (in 2011) (note that different constraints led to the same conclusions). We then scored a random number of larvae present on each tree as parasitized, calculated the parasitism rates, and regressed them against larval densities. We repeated this simulation 100 times. We found the upper 95th percentile of these regressions to be *r* = 0.38 (mean 0.01), i.e., well below our observed r of 0.79 and 0.42 (in 2010 and 2011, respectively). The observed relationships between larval densities and parasitism rate thus cannot be explained by a numerical bias.

Bird predation was quantified during spring 2011 using dummy larvae, i.e., artificial inedible plasticine larvae. Dummy larvae of two colors and shapes were designed to imitate the most abundant lepidopteran larvae in our study: dummy larvae of 3–4 cm length and 3–4 mm diameter were made either in green and with a straight shape (mimicking Tortricidae larvae) or in brown with an omega shape (mimicking Geometridae larvae). Three dummy larvae of each type were placed randomly on six different branches of each focal oak. Green-straight dummy larvae were positioned on leaves and brown-omega dummy larvae were directly positioned on branches. Dummy larvae were exposed for three weeks between the two samplings of ectophagous lepidopteran larvae in 2011 (see above). Due to the excessive height of even their lowest branches, it was not possible to put dummy larvae on two trees. Bird damage was defined as marks of a shape consistent with a bird beak and not explicable by other impacts such as scratching by nearby branches. The presence/absence of such marks on dummy larvae was recorded every three days with a total of four measurements. Damaged dummy larvae were replaced each time with intact ones, and consequently, the number of dummy larvae per tree remained the same during the whole experiment. Bird predation on a given tree was defined as the number of dummy larvae observed with bird damage, regardless of the amount of bird damage per larva, and summed for the entire period of the survey. Green dummy larvae were very seldom attacked compared to brown (11 vs. 67 times, respectively) probably because of their position on the leaves. We therefore only used the number of brown dummy larvae in our analyses. The effect of phylogenetic isolation remains unchanged and insignificant (see “Results” below) when accounting for green and brown dummy larvae together.

It has been suggested that bird predators learn to avoid dummy larvae, resulting in a possible underestimation of bird predation on true larvae (e.g., Aust and Steurer 2013). More critically, if learning to avoid dummy larvae happened more rapidly in some environments than in others (for instance, because birds are more stationary), this would bias the comparison of predation rates between environments. We hence tested the effect of phylogenetic isolation of host trees on bird predation of brown larvae based only on the earliest recording date, prior to a possible learning effect. These tests led to the same conclusion on the relationship between phylogenetic isolation and bird predation, based on all recording dates (for the 1st sampling date *df* = 18; *t* = −0.38; *P* = 0.70; *r* ^2^ = 8 × 10^−3^; for the 2nd sampling date *df* = 18; *t* = 0.62; *P* = 0.53; *r* ^2^ = 0.02; for the 3rd sampling date *df* = 18; *t* = 1.00; *P* = 0.32; *r* ^2^ = 0.05). The learning patterns of bird predators may also lead to more similar predation levels on more proximate trials (e.g., Remmel and Tammaru 2009). However, we found that predation levels on proximate trees (within pairs) were no more similar than predation levels on distant trees (between pairs). Overall, there is no evidence for a possible learning effect operating on more isolated but not on less isolated trees (or the inverse).

### Insect herbivory

Insect herbivory was measured as leaf damage in early September 2010 and 2011, about one month before leaf fall. Almost all leaf damage on oaks is caused prior to August (Rinker and Lowman 2004, p. 377). In 2010 and 2011, on each of the 22 crowns, 40 leaves were sampled on 15 September, 20 leaves from an upper stratum and 20 from a lower stratum, with both strata being sheltered from direct exposure to the sun. A total of 880 leaves were sampled. In both years, leaf damage was estimated with a 1 × 1-cm^2^ dot grid. The percentage of leaf damage was quantified as the number of dots covering the damaged parts of leaves relative to the number of dots covering an entire, undamaged leaf of the same size. No distinctions were made between feeding guilds (i.e., leaf chewers, leaf skeletonisers, leaf miners). Then, we calculated a mean percentage of damaged leaf area across the 40 leaves collected per tree. It ranged from 1 to 14 % in 2010, and from 1 to 12 % in 2011. Our estimate of herbivory is based solely on the remaining leaves, and might underestimate herbivory if highly grazed leaves were shed prior to our scoring. However, even moderately grazed leaves were rare: overall herbivory rates per crown were always ≤20 %, and 97 and 99 % of the individual leaves were grazed for <50 % in 2010 and 2011, respectively. Moreover, both herbivory and herbivore density declined with phylogenetic isolation, despite being recorded at very different moments in autumn and spring; respectively. Finally, herbivory declined with phylogenetic isolation in all years despite different absolute levels of herbivory. Overall, this indicates that bias due to sampling date is unlikely.

### Statistical analysis

For both types of enemy pressure (parasitism and bird predation rate), we applied the following procedure. We firstly used simple regression analyses to test *whether* parasitism rate is affected by phylogenetic isolation and other possible variables having a direct effect, such as ectophagous Lepidoptera density, insect herbivory, budburst phenology, canopy diversity, spatial distance to the next oaks, and the various physical and chemical characters recorded. Neither phylogenetic isolation nor any of the other variables were significantly related to bird predation pressure, and the analyses of bird predation pressure were terminated at this stage. In contrast, phylogenetic isolation, as well as some other variables, were significantly related to parasitism rate. We hence tried to identify *how* phylogenetic isolation affects parasitism. To identify variables having a direct effect on parasitism rate we used multiple regression analysis including phylogenetic isolation and the alternative variables that had been scored as significant in simple regression analyses, i.e., ectophagous Lepidoptera density, insect herbivory, and phenology. In these analyses, invoking multiple independent variables, we selected the model that best predicted the respective dependent variable using AIC_c_ as a criterion, as recommended by Johnson and Omland (2004) for small sample size or when the number of free parameters (i.e., independent variables), *P*, exceeds *n*/40, with *n* as sample size. Note that prior to these analyses, data were center-reduced (i.e., minus the mean and divided by the SD) in order to calculate standardized regression coefficients that permit us to estimate the effect size of each variable relative to the others. From these analyses we inferred the existence of a direct effect of phylogenetic isolation on parasitism rate when phylogenetic isolation was included, and scored as significant, in the best model to predict parasitism rate. To identify a possible indirect effect of phylogenetic isolation on parasitism rate, we related the phylogenetic isolation of oaks to the three above-mentioned variables having a possible direct effect on parasitism (ectophagous Lepidoptera density, herbivory, phenology), accounting for the physical and chemical characteristics of the oaks and the structure of the ambient canopy as alternative, indirect drivers. We applied the same model-selection procedures as described above for direct effects and inferred an indirect effect of phylogenetic isolation on parasitism rate when phylogenetic isolation was included and scored as significant in the best model to predict one variable having a direct effect on parasitism rate. See Appendices S8 and S9 for the results of this model selection to identify direct and indirect effects. We note that a path analyses (identifying direct and indirect effects, but without model selection) led to the same conclusions and is hence not presented.

2011 differed strongly from 2010: higher parasitism rate, lower density of ectophagous Lepidoptera, earlier phenology, and lower herbivory (ANOVA: *df* = 41; 1, *F* = 7.20, *P* = 0.01; *df* = 41; 1, *F* = 3.80, *P* = 0.05; *df* = 41; 1, *F* = 8.95, *P* = 4 × 10^−3^; *df* = 41; 1, *F* = 3.65, *P* = 0.06, respectively). We hence analyzed the data of 2010 and 2011 separately. However, analyzing the data of 2010 and 2011 together and accounting for “year” as a co-variable led to the same conclusion and is hence not presented.

We always found the residuals of our multiple regression analyses to approach normality and homoscedasticity, and hence we used a normal error distribution. All statistical analyses were performed using Statistica Version 9.0 (Statsoft, Maisons-Alfort, France). AIC and AIC_c_ were calculated with R version 2.9.2 (R Development Core Team 2011).

## Results

### Whether phylogenetic isolation affects parasitism rate

**Fig. 1:**
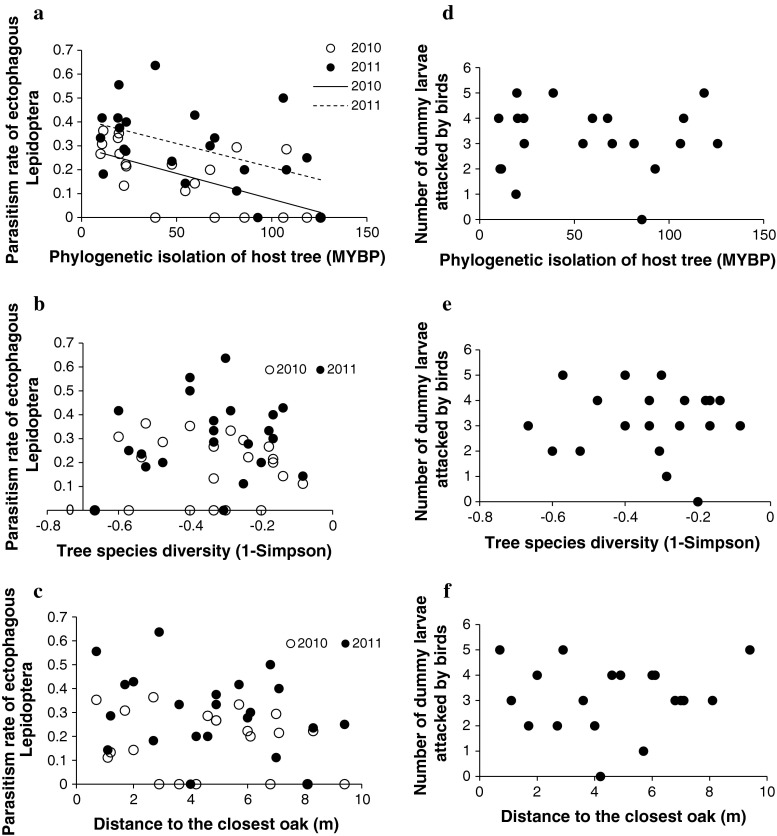
**a** Relationships between phylogenetic isolation of focal oak trees from neighboring trees and parasitism rate of ectophagous Lepidoptera in 2010 and 2011; (2010) *df* = 19, *t* = −3.48, *P* = 2 × 10^−3^, *r* ^2^ = 0.39; (**a** 2011) *df* = 20, *t* = −2.38, *P* = 0.02, *r* ^2^ = 0.22). **b** Relationships between diversity of the surrounding canopy and parasitism rate of ectophagous Lepidoptera in 2010 (*df* = 19; *t* = −9 × 10^−3^; *P* = 0.99; *r* ^2^ = 4 × 10^−6^); or 2011 (*df* = 20; *t* = 1.02; *P* = 0.31; *r* ^2^ = 0.04). **c** Relationships between distance to the closest conspecific host tree and parasitism rate of ectophagous Lepidoptera in 2010 (*df* = 19; *t* = −0.91; *P* = 0.37; *r* ^2^ = 0.04) or 2011 (*df* = 20; *t* = −1.28; *P* = 0.31; *r* ^2^ = 0.07). **d** Relationships between phylogenetic isolation of focal oak trees from neighboring trees and bird predation on ectophagous Lepidoptera in 2011 (*df* = 18, *r* ^2^ = 2 × 10^−3^, *t* = 0.20, *P* = 0.83). **e** Relationships between diversity of the surrounding canopy and bird predation on ectophagous Lepidoptera in 2011 (*df* = 18; *t* = −0.30; *P* = 0.76; *r* ^2^ = 5 × 10^−3^). **f** Relationships between distance to the closest conspecific host tree and bird predation on ectophagous Lepidoptera in 2011 (*df* = 18; *t* = 0.24; *P* = 0.81; *r* ^2^ = 3 × 10^−3^)

The parasitism rate ranged from 0 to 36 % in 2010 and from 0 to 63 % in 2011. In both years, parasitism rates are significantly lower on more phylogenetically isolated trees (see Fig. 1a, b; Tables 1, 2). In contrast, parasitism rate was not related to spatial distance from the next oak or to species diversity and species richness of the surrounding canopy (see Fig. 1c, d; Tables 1, 2).

**Table 1:** Variables that may have directly controlled the parasitism rate in 2010

Variable	*df*	*t* statistics	*P*	*r* ^2^
Phylogenetic isolation	19	−3.48	2 × 10^−3^	0.39
Ectophagous Lepidoptera density	19	5.62	2 × 10^−5^	0.62
Insect herbivory	19	1.79	0.08	0.14
Budburst phenology	19	−3.32	3 × 10^−3^	0.36
Diversity of the surrounding canopy	19	−9 × 10^−3^	0.99	4 × 10^−6^
Distance to the closest oak tree	19	−0.91	0.37	0.04
Species richness of the surrounding canopy	19	−0.54	0.58	0.01

Simple regression analyses testing the effect of phylogenetic isolation, diversity of the surrounding canopy, distance to the closest oak, insect herbivore density, insect herbivory, and budburst phenology

**Table 2:** Variables that may have directly controlled the parasitism rate in 2011

Variable	*df*	*t* statistics	*P*	*r* ^2^
Phylogenetic isolation	20	−2.38	0.02	0.22
Ectophagous Lepidoptera density	20	2.08	0.05	0.17
Insect herbivory	20	1.52	0.14	0.10
Budburst phenology	20	−1.96	0.06	0.16
Diversity of the surrounding canopy	20	1.02	0.31	0.04
Distance to the closest conspecific host tree	20	−1.28	0.31	0.07
Species richness of the surrounding canopy	20	−1.02	0.31	0.04

Simple regression analyses testing the effects of phylogenetic isolation, insect herbivore density, diversity of the surrounding canopy, distance to the closest oak, insect herbivory, and budburst phenology

### How phylogenetic isolation affects parasitism rate: direct and indirect effects

**Fig. 2:**
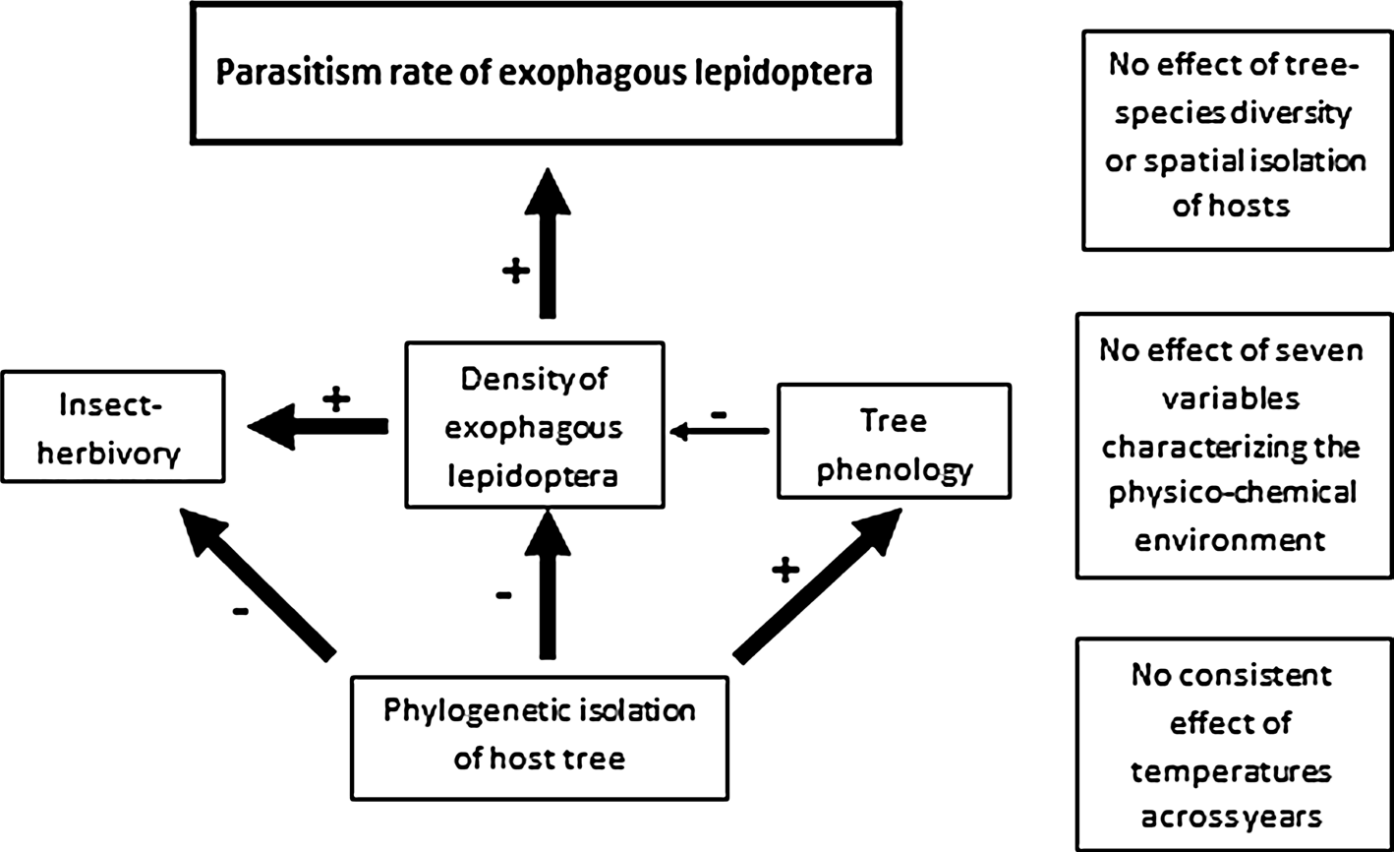
Figure representing the most probable link between variables that explain enemy pressure due to parasitoids in 2010 and 2011 based on the different analyses described in the “Methods”. The sign of each univariate relationship is indicated on *each arrow*. Tree phenology is the date of budburst. Bird predation is not presented, as it was not significantly related to any of the independent variables in simple or in multiple regression analysis

The parasitism rate in both 2010 and 2011 increased significantly with the density of ectophagous Lepidoptera, decreased with delayed budburst phenology (Table 1), was independent of the physico-chemical environment (Appendix S5) and, in 2010, tended to increase with herbivory (*P* = 0.08 vs. *P* = 0.14 in 2011). Phylogenetic isolation had no significant effect on the parasitism rate any more once Lepidoptera density, or herbivory or phenology, or any combination of these variables were included in the same model, both in 2010 and 2011. Lepidoptera density, in contrast, remained significant in 2010 (Appendix S8). Phylogenetic isolation, however, remained the most significant variable explaining Lepidoptera density in 2010 and 2011, even when accounting for characteristics of the physico-chemical environment, as well as tree species diversity, tree species richness, or distance to the next oak [Appendices S4, S5 and S9, except for temperature in 2011 (Appendix S9), which is correlated itself with phylogenetic isolation (Appendix S5)]. Also, phylogenetic isolation remains the most significant variable: in 2010 it best explains budburst or insect herbivory (indirectly via Lepidoptera), and in 2011 it explains insect herbivory and budburst (via temperature, Appendices S5 and S9). Therefore, for 2011 it is not possible to separate the direct and indirect effect of phylogenetic isolation on parasitism rate due to stronger colinearity with herbivore abundance, herbivory, and budburst. But phylogenetic isolation has at least an indirect negative effect on parasitism rate in both 2010 and 2011, mediated by a reduced Lepidoptera density (see Fig. 2).

We present a supplementary analysis focusing on galls and their parasitoids in Appendix S10. In short, these analyses show that the potential enemy pressure declined with the phylogenetic isolation of host trees per se, as well as with the parallel decline in leaf damage by non-galling insects, but not with the density of galls, thus indicating a direct effect of phylogenetic isolation.

### Testing whether phylogenetic isolation effects bird predation rate

Bird predation (tested in 2011) was not affected by phylogenetic isolation, canopy diversity, or spatial distance (Table 3), physico-chemical environment (Appendix S5), or any combination thereof (Appendix S9). Hence, no model-selection procedures were performed.

**Table 3:** Variables that may have directly controlled bird predation in 2011

Variable	*df*	*t* statistics	p	*r* ^2^
Phylogenetic isolation of host plant	18	−0.20	−0.83	2 × 10^−3^
Ectophagous Lepidoptera density	18	0.82	0.41	0.03
Insect herbivory	18	−0.11	0.91	0.02
Budburst phenology	18	−1.02	0.31	0.05
Diversity of the surrounding canopy	18	−0.30	0.76	5 × 10^−3^
Distance to the closest conspecific host tree	18	0.24	0.81	3 × 10^−3^
Species richness of the surrounding canopy	18	−0.67	0.50	0.02

Simple linear regression analyses testing the effects of phylogenetic isolation, insect herbivore density, diversity of the surrounding canopy, distance to the closest oak, insect herbivory, and budburst phenology on bird predation of ectophagous Lepidoptera in 2011

## Discussion

### Effect of phylogenetic isolation on enemy pressure

Our results show that, just like spatial fragmentation, the phylogenetic isolation of trees can negatively affect the enemy pressure faced by insect herbivores and hence increase their fitness. Phylogenetically isolated plant hosts might thus play a similar role as geographically isolated hosts, i.e., hosts in their introduced ranges, on which insect herbivores might indeed escape their natural enemies (Harvey et al. 2010).

However, it appears that the magnitude of this effect varies greatly with the degree of specialization of natural enemies. We found that insect herbivores on phylogenetically isolated host oak trees were released from the pressure of specialist enemies, i.e., parasitoids, but not from that of generalist enemies, i.e., birds. The effect of phylogenetic isolation on enemy pressure by parasitoids appears to be even stronger than the effect of spatial isolation of the host or of plant-community diversity. Even though parasitism rates can be highly variable between years, parasitism rates were related and unrelated to essentially the same factors in 2010 and 2011. This release of insect herbivores from specialist enemies on phylogenetically isolated host trees likely resulted from an indirect effect mediated via reduced insect herbivore density (see also, Yguel et al. 2011): with decreasing phylogenetic isolation of plants, the increase of insect herbivore density leads to the over-proportional attraction and, ultimately, the aggregation of parasitoids. The well-known phenomenon of density-dependent attack by natural enemies (Crawley 1983) therefore has previously unknown consequences for the fitness of herbivores that succeed in tracking host plants in a phylogenetically distant neighborhood.

We note that we observed equivalent overall relationship patterns in galls and their parasitoids (i.e., Chalcidoidea): the former increased with increasing phylogenetic isolation of oaks, while the latter declined in density (see Appendix S10). Interestingly, here, the phylogenetic isolation of trees appeared to have not only an indirect but also a direct effect on parasitoids, while parasitoids do not seem to respond to the abundance of their galls hosts but instead to herbivory by non-host herbivores.

### Limitations of our study

The effects of phylogenetic isolation considered to be “indirect” here are those mediated by known factors controlling the attack rates of natural enemies, i.e., prey abundance and phenology of the prey’s host or host microclimate. However, we are aware that millions of years of evolutionary distance are not important by themselves, but only via increased evolutionary differentiation of traits between hosts. This concerns traits in general and, more specifically, traits that have co-evolved with the respective herbivore/parasitoid faunas. In that very strict sense, phylogenetic isolation always has only an indirect effect.

The limited sample size (*N* = 22 trees) and the correlation between phylogenetic isolation, insect herbivory, and insect herbivore density in 2010 (Appendix S9) made it difficult to fully evaluate their relative importance to the parasitism rate of ectophagous Lepidoptera (Petraitis et al. 1996). The results of multiple regression analyses thus need to be considered carefully. Consequently, to fully identify the relative importance of each factor on the parasitism rate, new controlled experiments with a larger sample size and independent manipulation of herbivore density and phylogenetic isolation would be required. On the other hand, the robustness of all our major results to the variation between years increases our confidence in the conclusions: parasitism rate depends on phylogenetic distance, not on tree species diversity or distance to the next oak. Moreover, phylogenetic isolation operates primarily indirectly via a reduction of herbivory and, hence, parasitoid attraction.

A reduced parasitism rate on phylogenetically isolated trees might also result from changes in species richness or composition of parasitoids. However, the number of sampled parasitoids was too small to test the effect of richness on parasitism rate independently of density. Additional sampling would be needed to check whether the effect of phylogenetic isolation on parasitism rate could be explained by a change in the diversity of specialist enemies.

### Why phylogenetic isolation of host plants reduces enemy pressure by parasitoids

For a parasitoid, two characteristics are essential for finding its insect herbivore host: dispersal capacity and host-seeking ability. Parasitoids can disperse over kilometers (Santos et al. 2011), while in our study, the phylogenetically isolated and non-isolated trees were separated by <150 m (see “Methods”). Thus, dispersal capacity is clearly not a limiting factor. The host-seeking ability of parasitoids is known to be essentially based on chemical cues (Dicke and Van Loon 2000; Rott et al. 2005; Williams et al. 2007). Particularly, host-plant odors, volatile compounds released by damaged leaves (Thaler 1999) or by insect herbivores themselves, are known to attract parasitoids and then to enhance enemy pressure (Du Merle 1988; Mills 1993; Dicke and Van Loon 2000).

Each of these three factors might indeed play a role in our study system. Firstly, from the point of view of parasitoids, a host plant that is phylogenetically isolated is likely to be surrounded by non-suitable hosts. Plant odors released by distantly related hosts may act as a repellent or barrier for parasitoids and hide suitable hosts, as it has already been described for insect herbivores (Jactel et al. 2011). Thus, phylogenetic isolation may have a direct effect on host-seeking by parasitoids, independently of the effect of insect herbivore density or insect herbivory. However, our analysis including insect herbivory as a covariable indicates that no such direct effect of phylogenetic isolation exists. Secondly, the decrease in insect-herbivore density and in insect herbivory, which were caused by the phylogenetic isolation of host plants (Yguel et al. 2011), might also have decreased the olfactory attractiveness of the host plant for parasitoids, and subsequently, decreased the parasitism rate (Crawley 1983). Furthermore, the seeking of insect herbivores by parasitoids might be facilitated by the spatial clustering of insect hosts (Stireman and Singer 2003). Clustered groups of herbivore hosts may be more easily located than spatially isolated herbivore individuals, and within a group an individual insect herbivore is simple to locate for a parasitoid. The decrease in insect herbivore density on phylogenetically isolated host trees may have resulted in a reduced clustering of insect herbivores that would then render herbivore-host finding more difficult and costly for parasitoids (density-dependent attack rate, Crawley 1983). In these latter cases, the effect of phylogenetic isolation would be indirectly mediated by a decline in insect herbivore density and insect herbivory. This is exactly what we observed in 2010 and in 2011. Overall, increasing search costs due to difficulties in locating isolated host plants, and sparse and un-clustered herbivore hosts should lead to decreased aggregation of parasitoids on isolated host plants (see also Bernstein et al. 1991). However, we reiterate that the experimental manipulation of each potential cause of parasitism rate would be required to fully evaluate their relative importance.

### Why phylogenetic isolation of host plants does not decrease predation pressure by birds

Contrary to parasitoids, bird predation pressure on ectophagous Lepidoptera was not affected by the phylogenetic isolation of host trees. There are several possible reasons for this. Firstly, the foraging range of predatory birds is often longer than the distance separating strongly and weakly phylogenetically isolated trees (i.e., <150 m; e.g., in Tremblay et al. 2005). Secondly, given that bird predation was not significantly related to insect herbivory or insect herbivore density, the bird predators might not have been able to detect olfactory cues emitted by oaks attacked by insect herbivores (Mantyla et al. 2008). Thirdly, insectivorous forest birds are often less specialized on particular insect herbivore species than insect parasitoids: birds were probably also feeding on insect herbivores living on non-oak trees surrounding the oaks in our experimental site, so that phylogenetically isolated oak trees may not have been perceived by predatory birds as functionally isolated resource patches.

### Implications for pest control

The concept of associational resistance suggests that the identity of surrounding plants may result in lower herbivory on a focal plant (Atsatt and Odowd 1976; Barbosa et al. 2009). One mechanism that has been proposed to explain associational resistance is the natural enemy hypothesis, which states that plant diversity can favor the abundance of natural enemies and their effectiveness to control herbivores (e.g., Letourneau 1987). However, here, we show that on a tree surrounded by phylogenetically distant trees, an insect herbivore will experience a lower, rather than higher, enemy pressure. We may hence speculate that on such phylogenetically isolated host plants, or in phylogenetically diverse plant communities, herbivores are less controlled by natural enemies and plants might suffer higher attacks by insect herbivores. But this speculation only holds if insect herbivores themselves are not limited by phylogenetic isolation. In our study system, that was probably the case for galls (Appendix S10), but not for ectophagous Lepidoptera (Yguel et al. 2011). In contrast to specialist enemies, generalist predators (e.g., birds) might not be affected by the phylogenetic isolation of host trees. Hence, generalist enemies could be more useful than specialist enemies in controlling herbivorous pest insects on phylogenetically isolated host plants, and hence in phylogenetically diverse plant communities. Obviously, this requires that such generalist enemies are capable of exerting a top-down control sufficient enough to reduce pest populations below an economically tolerable level, which remains to be tested. In consequence, the associational resistance of plants due to a phylogenetically diverse ambient community might become associational susceptibility if insect herbivores are extreme specialists (here: galls) or if their enemies are specialists (here: parasitoids); the former are not limited by the phylogenetic distance of the neighbors, while the latter are. This hypothesis remains to be tested in future research.

### Evolutionary implications for phylogenetically isolated host trees and their interacting species

Phylogenetic distance from the neighborhood can be interpreted in terms of niche evolution. If niches are more similar among closely related than among distantly related species (as demonstrated for the present flora by Prinzing et al. 2001, niche conservatism; Wiens et al. 2010), then a given niche will be dominated by closely related neighbors. For instance, fish have conserved an aquatic niche and most vertebrates in an aquatic niche are fish. An individual in a closely related neighborhood (a fish among fish) is likely to be in its ancestral neighborhood. An individual in a distantly related neighborhood (a mammal among fish) is likely one that has left its ancestral neighborhood, that is, one that has broken with niche conservatism. The present study shows that trees breaking with niche conservatism tend to be hosts on which insect herbivores can escape their enemies.

Overall, the enemy release for the phylogenetically isolated host plants (Yguel et al. 2011) is transmitted to its insect herbivores that also benefit from a release from their specialist enemies. This transmission of enemy release through the trophic chain may originate from similarities in host-seeking behavior between insect herbivores and parasitoids, i.e., attraction by host-plant odors. This effect may be combined with phylogenetic conservatism in host-seeking behavior, with closely related insect herbivores or parasitoids seeking closely related host plants and being repelled by distantly related plants.

Our results imply that the benefits of phylogenetic isolation for a host tree due to reduced enemy pressure (Yguel et al. 2011) are mitigated by enemy release of its insect herbivores. In particular, highly specialist enemies of plants (like galls) may be capable of tracking their host plants in any neighborhood (see also Chust et al. 2007), and thereby escape their own enemies affected by the phylogenetic isolation of host plants. This might potentially invert the release for the plant from highly specialized enemies, and thus would result in fitness loss of the host plants in phylogenetically distant neighborhoods. Therefore, in the very specific case of a plant being attacked mainly by highly specialized herbivores and these herbivores suffering strong enemy pressure, we might expect that the plant suffers rather than profits from leaving the ancestral niche. This would reinforce the niche conservatism of host plants. Overall, for plants, predicting the evolutionary consequences of phylogenetic isolation should take into account the effect of such phylogenetic isolation across the entire trophic chain.

Our results also imply that the selective pressure of parasitoids on insect herbivores may change with the phylogenetic isolation of their host plants. Lower and less specialized enemy pressure on phylogenetically isolated host trees might increase the fitness of insect herbivores. A possible consequence is the divergent selection of individuals reaching phylogenetically isolated hosts versus individuals staying on non-phylogenetically isolated hosts. The former might, for instance, be selected for high host-seeking capacities, and the latter for efficient enemy defense strategies. Such evolutionary differentiation between insect-herbivore lineages on phylogenetically isolated and non-isolated trees of the same species would be consistent with recent concepts of ecological speciation in insect herbivores following plant evolution (Singer and Stireman 2005; Nyman 2010). Phylogenetically isolated hosts could, in fact, be viewed in our study as an environmental island sensu (Ackerly 2003) that “influences the functional attributes and environmental tolerance of colonizers and subsequent pressures and opportunities for adaptive evolution”, due to dispersal limitation and reduced parasitism pressure. Further analyses based on genetic and quantitative trait comparisons of the different populations of insect herbivores found on phylogenetically isolated and non-isolated host trees are now required to test these hypotheses on the possible evolutionary differentiation of insect herbivores.

## Electronic supplementary material

Below is the link to the electronic supplementary material.

Online Appendices (DOC 115 kb)

## References

[CR1] Ackerly DD (2003). Community assembly, niche conservatism, and adaptive evolution in changing environments. Int J Plant Sci.

[CR2] Angiosperm Phylogeny Group (2009). An update of the Angiosperm Phylogeny Group 43 classification for the orders and families of flowering plants: APG III. Bot J Linn Soc.

[CR3] Atsatt PR, Odowd DJ (1976). Plant defense guilds. Science.

[CR4] Aust U, Steurer MM (2013). Learning of an oddity rule by pigeons in a four-choice touch-screen procedure. Anim Cogn.

[CR5] Barbosa P, Hines J, Kaplan I, Martinson H, Szczepaniec A, Szendrei Z (2009) Associational resistance and associational susceptibility: having right or wrong neighbors. In: Annual review of ecology, evolution, and systematics, vol 40. Annual Reviews, Palo Alto, pp 1–20

[CR6] Bernstein C, Kacelnik A, Krebs JR (1991). Individual decisions and the distribution of predators in a patchy environment. 2. The influence of travel costs and structure of the environment. J Anim Ecol.

[CR7] Bertheau C, Brockerhoff EG, Roux-Morabito G, Lieutier F, Jactel H (2010). Novel insect-tree associations resulting from accidental and intentional biological ‘invasions’: a meta-analysis of effects on insect fitness. Ecol Lett.

[CR8] Bruckmann SV, Krauss J, van Achterberg C, Steffan-Dewenter I (2011). The impact of habitat fragmentation on trophic interactions of the monophagous butterfly *Polyommatus coridon*. J Insect Conserv.

[CR9] Castagneyrol B, Jactel H (2012). Unraveling plant–animal diversity relationships: a meta-regression analysis. Ecology.

[CR10] Chust G, Garbin L, Pujade-Villar J (2007). Gall wasps and their parasitoids in cork oak fragmented forests. Ecol Entomol.

[CR11] Crawley M (1983). Herbivory: the dynamics of plant–animal interactions.

[CR12] Dicke M, Van Loon JJA (2000). Multitrophic effects of herbivore-induced plant volatiles in an evolutionary context. Entomol Exp Appl.

[CR13] Du Merle P (1988) Phenological resistance of oaks to the green oak leafroller, *Tortrix viridana* (Lepidoptera: Tortricidae). In: Mattson WJ, Levieux J, Bernard-Dagan C (eds) Mechanism of woody plant defenses against insects. Springer, Berlin, p 416

[CR14] Futuyma DJ, Agrawal AA (2009). Macroevolution and the biological diversity of plants and herbivores. Proc. Natl. Acad. Sci. USA.

[CR15] Gilbert GS, Webb CO (2007). Phylogenetic signal in plant pathogen-host range. Proc Natl Acad Sci USA.

[CR16] Gossner MM, Chao A, Bailey RI, Prinzing A (2009). Native fauna on exotic trees: phylogenetic conservatism and geographic contingency in two lineages of phytophages on two lineages of trees. Am Nat.

[CR17] Hairston NG, Smith FE, Slobodkin LB (1960). Community structure, population control, and competition. Am Nat.

[CR18] Harvey JA, Bukovinszky T, van der Putten WH (2010). Interactions between invasive plants and insect herbivores: a plea for a multitrophic perspective. Biol Conserv.

[CR19] Holt RD, Lawton JH, Polis GA, Martinez ND (1999). Trophic rank and the species–area relationship. Ecology.

[CR20] Jactel H, Birgersson G, Andersson S, Schlyter F (2011). Non-host volatiles mediate associational resistance to the pine processionary moth. Oecologia.

[CR21] Johnson JB, Omland KS (2004). Model selection in ecology and evolution. Trends Ecol Evol.

[CR22] Kondoh M (2003). Habitat fragmentation resulting in overgrazing by herbivores. J Theor Biol.

[CR23] Koricheva J, Vehvilainen H, Riihimaki J, Ruohomaki K, Kaitaniemi P, Ranta H (2006). Diversification of tree stands as a means to manage pests and diseases in boreal forests: myth or reality?. Can J For Res Rev Can Res For.

[CR24] Kruess A, Tscharntke T (1994). Habitat fragmentation, species loss, and biological-control. Science.

[CR25] Le Corff J, Marquis RJ, Whitfield JB (2000). Temporal and spatial variation in a parasitoid community associated with the herbivores that feed on Missouri Quercus. Environ Entomol.

[CR26] Legendre P, Dale MRT, Fortin MJ, Casgrain P, Gurevitch J (2004). Effects of spatial structures on the results of field experiments. Ecology.

[CR27] Letourneau DK (1987). The enemies hypothesis—tritrophic interactions and vegetational diversity in tropical agroecosystems. Ecology.

[CR28] Mantyla E (2008). From plants to birds: higher avian predation rates in trees responding to insect herbivory. PLoS ONE.

[CR29] Mills NJ (1993). Species richness and structure in the parasitoid complexes of tortricoid hosts. Ecology.

[CR30] Nyman T (2010). To speciate, or not to speciate? Resource heterogeneity, the subjectivity of similarity, and the macroevolutionary consequences of niche-width shifts in plant-feeding insects. Biol Rev.

[CR31] Ozanne CMP, Speight MR, Hambler C, Evans HF (2000). Isolated trees and forest patches: patterns in canopy arthropod abundance and diversity in *Pinus sylvestris* (Scots pine). For Ecol Manag.

[CR32] Petraitis PS, Dunham AE, Niewiarowski PH (1996). Inferring multiple causality: the limitations of path analysis. Funct Ecol.

[CR33] Prinzing A, Durka W, Klotz S, Brandl R (2001). The niche of higher plants: evidence of phylogenetic conservatism. Proc R Soc B Biol Sci.

[CR34] R Development Core Team (2011) A language and environment for statistical computing. R Foundation for Statistical Computing, Vienna. ISBN 3-900051-07-0. http://www.r-project.org/

[CR35] Remmel T, Tammaru T (2009). Size-dependent predation risk in tree-feeding insects with different colouration strategies: a field experiment. J Anim Ecol.

[CR36] Rinker HB, Lowman MD (2004) Insect herbivory in tropical forests. In: Lowman MD, Rinker HB (eds) Forest canopies, 2nd edn, chap 18. Academic Press, New York, pp 359–386

[CR37] Rosenzweig ML (1995). Species diversity in space and time.

[CR38] Rott AS, Hackermann J, Brand N, Vallat A, Dorn S (2005). Parasitoid exploitation of the seasonal variation in host plant volatile emission for herbivore location. Entomol Exp Appl.

[CR39] Rousset O, Lepart J (2000). Positive and negative interactions at different life stages of a colonizing species (*Quercus humilis*). J Ecol.

[CR40] Santos AMC, Fontaine C, Quicke DLJ, Borges PAV, Hortal J (2011). Are island and mainland biotas different? Richness and level of generalism in parasitoids of a microlepidopteran in Macaronesia. Oikos.

[CR41] Singer MS, Stireman JO (2005). The tri-trophic niche concept and adaptive radiation of phytophagous insects. Ecol Lett.

[CR42] Southwood TRE, Wint GRW, Kennedy CEJ, Greenwood SR (2004). Seasonality, abundance, species richness and specificity of the phytophagous guild of insects on oak (Quercus) canopies. Eur J Entomol.

[CR44] Stireman JO, Singer MS (2003). Determinants of parasitoid–host associations: insights from a natural tachinid-lepidopteran community. Ecology.

[CR45] Thaler JS (1999). Jasmonate-inducible plant defences cause increased parasitism of herbivores. Nature.

[CR46] Tremblay I, Thomas D, Blondel J, Perret P, Lambrechts MM (2005). The effect of habitat quality on foraging patterns, provisioning rate and nestling growth in Corsican blue tits *Parus caeruleus*. Ibis.

[CR47] Vialatte A (2010). Phylogenetic isolation of host trees affects assembly of local Heteroptera communities. Proc R Soc B Biol Sci.

[CR48] Violle C, Nemergut DR, Pu ZC, Jiang L (2011). Phylogenetic limiting similarity and competitive exclusion. Ecol Lett.

[CR49] Wesolowski T, Rowinski P (2008). Late leaf development in pedunculate oak (Quercus robur): an antiherbivore defence?. Scand J For Res.

[CR51] Wiens JJ, Ackerly DD, Allen AP, Anacker BL, Buckley LB, Cornell HV, Damschen EI, Davies TJ, Grytnes JA, Harrison SP, Hawkins BA, Holt RD, McCain CM, Stephens PR (2010). Niche conservatism as an emerging principle in ecology and conservation biology. Ecol Lett.

[CR52] Williams IH, Frearson DJT, Barari H, McCartney A (2007). First field evidence that parasitoids use upwind anemotaxis for host-habitat location. Entomol Exp Appl.

[CR53] Yguel B (2011). Phytophagy on phylogenetically isolated trees: why hosts should escape their relatives. Ecol Lett.

