# Supplementary material for: Insect herbivores should follow plants escaping their relatives
Source: Oecologia. 2014 Jul 23;176(2):521–32. doi: 10.1007/s00442-014-3026-3 (PMC4161943; doi:10.1007/s00442-014-3026-3)
Supplement: Supplementary file 1 — Online Appendices (DOC 115 kb) [file 442_2014_3026_MOESM1_ESM.doc]

**Online Appendixes**

**Online Appendix S1.** Information on the Rennes Forest and on the species composition of the trees surrounding the focal oaks studied.

**Online Appendix S2.** Effect of tree pair and tree species on enemy pressure and on insect herbivores

**Online Appendix S3.** Definition of phylogenetic isolation and table of phylogenetic distances.

**Online Appendix S4.** Effect of tree diversity, species richness of the surrounding canopy and spatial isolation on ectophagous Lepidoptera density, on herbivory, and on budburst phenology.

**Online Appendix S5.** Effect of phylogenetic isolation on the physicochemical environment, and effect of the physicochemical environment on enemy pressure, ectophagous Lepidoptera density and herbivory

**Online Appendix S6.** Species composition of ectophagous Lepidoptera parasitoids in 2010 and 2011.

**Online Appendix S7.** Variables correlated to species richness of ectophagous Lepidoptera parasitoids in 2010 and 2011.

**Online Appendix S8** Model selection identifying variables directly affecting enemy pressure on ectophagous Lepidoptera (parasitism rate in 2010 and 2011, and bird predation in 2011).

**Online Appendix S9.** Model selection identifying variables controlling variables directly affecting parasitism rate, i.e. ectophagous Lepidoptera density, insect herbivory, and budburst phenology.

**Online Appendix S10.** Effect of phylogenetic isolation of host tree on the density of galls and their parasitoids.

**Online Appendix S1.** **Information on the Rennes Forest and on the species composition of the trees surrounding the focal oaks studied.**

The Forest of Rennes dates back to at least the 12th century. As with all forests in Western and Central Europe, this forest is under the influence of human activities, such as wood management and the effects of surrounding agricultural land use. The Forest of Rennes is split into parcels, mostly managed following the shelterwood cutting system (Borghetti & Giannini 2001). Each parcel is planted typically either with oak (*Quercus petraea* or *Q. robur*) or pine (*Pinus sylvestris*). As in numerous European temperate forests, the other main tree species in these parcels are *Abies alba, Alnus glutinosa, Betula pendula, Carpinus betulus, Castanea sativa, Corylus avellana, Fagus sylvatica*, *Ilex aquifolium*, *Malus sylvestris, Sorbus torminalis, Populus tremula, Prunus avium, Pyrus pyraster* , *Rhamnus frangula, Salix* *caprea, Tilia cordata* and *Ulmus minor*. All these species are native to Europe and were in contact with the focal oaks.

**Online Appendix S2. Effect of tree pair and tree species on enemy pressure and on insect herbivores**

We found that tree “pair” did not significantly affect parasitism rate of ectophagous Lepidoptera in 2010 and 2011 (ANOVA, d.f. =11, F =1.22 , P =0.37 in 2010 and ANOVA, d.f. =11, F =0.46, P =0.88 in 2011) or bird predation on brown dummy larvae (ANOVA, d.f. =9, F =1.39, P =0.31). Thus we pooled tree pair with the error term. As expected given the sampling design, we also found that *Q. petraea* vs. *Q. robur* had no significant impact on parasitism rate in 2010 and 2011 (ANOVA, d.f. =20 , F <0.01 , P =0.94 in 2010 and ANOVA, d.f. =20 , F 0.31 , P =0.58 in 2011) or on bird predation on dummy larvae in 2011 (ANOVA, d.f. =18, F =0.43 , P =0.51) nor bird predation on brown dummy larvae (ANOVA, d.f. =18, F =1.90, P =0.18).

**Online Appendix S3. Definition of phylogenetic isolation and table of phylogenetic distances**

Phylogenetic distance is the estimated time (in MYBP) since the evolutionary establishment of the clades of a given neighboring tree species and of oaks. These phylogenetic distances were taken from Vialatte et al. 2010 and Yguel et al. 2011. Note that this is not the most recent common ancestor, as this would give pines an extreme weight, given the extreme age of gymnosperms (probably more than 160 million years older than angiosperms as we know them today, Savard et al., 1994), and would essentially render our parameter a simple percentage of pines in the surroundings of the oaks. Rather, this is the age when both sister clades had established their particular characteristics as hosts for insects (i.e. phylogenetic crown-age of the younger of the two lineages and not stem age). See Methods for further explanations.

The table below gives phylogenetic distances in millions of years before present between oak and the other tree species in the communities. Distance corresponds to the smaller of the two phylogenetic tree crown ages of the two lineages involved (i.e. of oak and of the other tree species) at the corresponding phylogenetic rank (inferred from Magallon et al., 1999, Manos et al., 1999, Wikström et al., 2001, APG 2003, and 2009, Poinar et al., 2007). This table is extracted from Vialatte et al. 2010 and Yguel et al. 2011. See Methods for further explanations.

| Species | Phylogenetic rank of separation with oak | | | | | | distance |
| --- | --- | --- | --- | --- | --- | --- | --- |
| *Chamaecyparis* sp. | Spermatophytes | - | - | - | - | - | 140 |
| *Pinus sylvestris* | Spermatophytes | - | - | - | - | - | 140 |
| *Abies* sp. | Spermatophytes | - | - | - | - | - | 140 |
| *Ilex* sp. | Angiosperms | Asterids | - | - | - | - | 128 |
| *Tilia* sp. | Angiosperms | Rosids | Malvids | - | - | - | 89.5 |
| *Salix caprea* | Angiosperms | Rosids | Fabids | Malpighiales | - | - | 68 |
| *Populus tremula* | Angiosperms | Rosids | Fabids | Malpighiales | - | - | 68 |
| *Rhamnus* sp*.* | Angiosperms | Rosids | Fabids | Rosales | - | - | 58.5 |
| *Prunus* sp. | Angiosperms | Rosids | Fabids | Rosales | - | - | 58.5 |
| *Sorbus* sp | Angiosperms | Rosids | Fabids | Rosales | - | - | 58.5 |
| *Pyrus* sp. | Angiosperms | Rosids | Fabids | Rosales | - | - | 58.5 |
| *Malus* sp. | Angiosperms | Rosids | Fabids | Rosales | - | - | 58.5 |
| *Ulmus minor* | Angiosperms | Rosids | Fabids | Rosales | - | - | 58.5 |
| *Alnus glutinosa* | Angiosperms | Rosids | Fabids | Fagales | Betulaceae | - | 54 |
| *Corylus avellana* | Angiosperms | Rosids | Fabids | Fagales | Betulaceae | - | 54 |
| *Betula* sp. | Angiosperms | Rosids | Fabids | Fagales | Betulaceae | - | 54 |
| *Carpinus betulus* | Angiosperms | Rosids | Fabids | Fagales | Betulaceae | - | 54 |
| *Fagus sylvatica* | Angiosperms | Rosids | Fabids | Fagales | Fagaceae | Fagus | 40 |
| *Castanea sativa* | Angiosperms | Rosids | Fabids | Fagales | Fagaceae | Castanea | 40 |

**References:**

Angiosperm Phylogeny Group (2003) An update of the Angiosperm phylogeny group classification for the orders and families of flowering plants: APG II. Bot J Linn Soc 141: 399–436.

Angiosperm Phylogeny Group (2009) An update of the Angiosperm Phylogeny Group classification for the orders and families of flowering plants: APG III", Bot J Linn Soc 161: 105–121

Magallon S, Crabe PR, Herendeen PS (1999) Phylogenetic pattern, diversity, and diversification of eudicots. Ann Missouri Bot Garden 86: 1407–1419.

Manos PS, Doyle JJ, Nixon KC (1999) Phylogeny, biogeography, and processes of molecular differentiation in *Quercus* subgenus *Quercus* (Fagaceae). Mol Phyl Evol 12: 333–349.

Poinar G, Chambers KL, Buckley R (2007) *Eoepigynia burmensis* gen. and sp. nov., an early Cretaceous Eudicot flower (Angiospermae) in Burmese Amber. J Bot Res Inst Texas 1: 91–96.

Savard L, Li P, Strauss SH, Chase MW, Michaud M, Bousquet J (1994). Chloroplast and nuckear genes-sequences indicate late Pennsylvanian time for the last common ancestor of extant seed plants. Proc Natl Acad Sci USA 91: 5163-5167.

Vialatte A, Bailey RI, Vasseur C, Matocq A, Gossner MM, Everhart D, Vitrac X, Belhadj A, Ernoult A & Prinzing A (2010) Phylogenetic isolation of host trees affects assembly of local Heteroptera communities. Proc Biol Sci 277: 2227-2236.

Wikström, N, Savolainen, V & Chase, M W (2001) Evolution of the angiosperms: calibrating the family tree. Proc R Soc. Lond. B 268, 2211–2220.

**Online Appendix S4.** **Effect of tree diversity, species richness of the surrounding canopy and spatial isolation on ectophagous Lepidoptera density, on herbivory, and on budburst phenology**

Species diversity (i.e. 1-Simpson index) of the surrounding canopy had no effect on exophagous lepidoptera density (d.f.=20; t=0.37; P=0.70; r²=7*10^-3^; b*=0.08 in 2010; d.f.=20; t= 0.12; P=0.89; r²=8*10^-4^; b*=0.02 in 2011), herbivory (d.f.=20; t=0.49; P=0.62; r²=0.01; b*=-0.11 in 2010; d.f.=20; t= 0.22; P=0.82; r²=2*10^-3^; b*=0.05 in 2011). Budburst phenology was not affected significantly by species diversity of the surrounding canopy (d.f.=20; t=0.14; P=0.88; r²=1*10^-3^; b*=0.03 in 2010; d.f.=20; t= 1.11; P=0.27; r²=0.05; b*=0.24 in 2011). Besides, species diversity of the surrounding canopy was not significantly correlated to phylogenetic isolation (d.f.=20; t= -1.15; P=0.26; r²=0.06; b*=-0.24).

Tree species richness had no significant effect on exophagous Lepidoptera density (d.f.=20; t= -0.58; P=0.56; r²=0.01; b*=-0.12 in 2010; d.f.=20; t= -0.41; P=0.68; r²=8*10^-3^; b*=-0.09 in 2011) on herbivory (d.f.=20; t=-1.25; P=0.22; r²=0.7; b*=-0.27 in 2010 d.f.=20; t= -0.64; P=0.52; r²=0.02; b*=-0.14 in 2011) or on budburst (d.f.=20; t= 0.14; P=0.88; r²=1*10^-3^; b*=0.03 in 2010 ; d.f.=20; t= 1.87; P=0.07; r²=0.14; b*=0.38 in 2011). Besides, tree species richness was not significantly correlated to phylogenetic isolation (d.f.=20; t= 0.65; P=0.52; r²=0.02; b*=0.14).

Distance to the closest conspecific host tree also had no effect on exophagous Lepidoptera density (d.f.=20; t=-1.13; P=0.26; r²=0.06; b*=-0.24 in 2010; d.f.=20; t=-0.12; P=0.89; r²=8*10^-4^; b*=-0.02 in 2011) , herbivory (d.f.=20; t=-0.62; P=0.54; r²=0.01; b*=-0.13 in 2010; d.f.=20; t= -0.84; P=0.40; r²=0.03;b*=-0.18 in 2011). Budburst phenology was not affected significantly by distance to closest conspecific host tree (d.f.=20; t=1.05; P=0.30; r²=0.05; b*=0.23 in 2010; d.f.=20; t= 1.12; P=0.27; r²=0.05; b*=0.24 in 2011).

**Online Appendix S5.** **Effect of phylogenetic isolation on the physicochemical environment, and effect of the physicochemical environment on enemy pressure, ectophagous Lepidoptera density and herbivory**

Analyses for 2010 and 2011 contribute to explaining proximate and ultimate controls of enemy pressure on exophagous Lepidoptera. Analyses for 2006 contribute to explaining proximate and ultimate controls of parasite abundance on leave galls (see Appendix 10)

Table S5.a Effect of environmental parameters on the parasitism rate of ectophagous Lepidoptera in 2010 in simple regression analysis. Note that for technical reasons temperature and humidity were not recorded on one tree in March, April and on two trees in May. Thus sample size was not 21 but 20 or 19 trees.

|  | Effect on parasitism rate 2010 | | | |
| --- | --- | --- | --- | --- |
|  | Df | T | p | R² |
| crown size | 19 | -0,39 | 0.69 | 8*10^-3^ |
| crown volume | 19 | -0.47 | 0.64 | 0.01 |
| Temperature march | 18 | 0.75 | 0.46 | 0.03 |
| Humidity march | 18 | -1.01 | 0.32 | 0.05 |
| Temperature april | 18 | 0.71 | 0.48 | 0.02 |
| Humidity april | 18 | -0.56 | 0.58 | 0.01 |
| Temperature may | 17 | -0.17 | 0.85 | 1*10^-3^ |
| Humidity may | 19 | 1.41 | 0.17 | 0.09 |
| leaf C/N ratios | 19 | 0.75 | 0.46 | 0.02 |
| dry weight | 19 | 1.04 | 0.31 | 0.05 |

Table S5.b Effect of environmental parameters on the parasitism rate of ectophagous Lepidoptera in 2011 in simple regression analysis. Note that for technical reasons temperature and humidity were not recorded on 7 trees. Thus sample size was not 22 but 15 trees.

|  | Effect on parasitism rate 2011 | | | |
| --- | --- | --- | --- | --- |
|  | Df | T | p | R² |
| Crown size | 20 | -0.49 | 0.62 | 0.01 |
| Crown volume | 20 | -0.81 | 0.42 | 0.03 |
| Temperature march | 13 | 0.95 | 0.35 | 0.06 |
| Humidity march | 13 | 0.50 | 0.62 | 0.01 |
| Temperature april | 13 | 1.56 | 0.14 | 0.15 |
| Humidity april | 13 | 0.25 | 0.80 | 5*10^-3^ |
| Temperature may | 13 | 0.26 | 0.79 | 5*10^-3^ |
| Humidity may | 13 | 0.48 | 0.63 | 0.01 |
| Leaf C/N ratios | NA | NA | NA | NA |
| Dry weight | NA | NA | NA | NA |

Table S5.c Effect of environmental parameters on bird predation on dummy larvae in 2011 in simple regression analysis. Note that for technical reasons temperature and humidity were not recorded on 7 trees. Thus sample size was not 22 but 13 trees.

|  | Effect on Bird predation rate | | | |
| --- | --- | --- | --- | --- |
|  | Df | T | p | R² |
| Crown size | 18 | 0.84 | 0.40 | 0.03 |
| Crown volume | 18 | 0.46 | 0.64 | 0.01 |
| Temperature march | 11 | 0.76 | 0.46 | 0.04 |
| Humidity march | 11 | 1.10 | 0,29 | 0.10 |
| Temperature april | 11 | 0.69 | 0.50 | 0.04 |
| Humidity april | 11 | 0.96 | 0.35 | 0.07 |
| Temperature may | 11 | 0.53 | 0.60 | 0.02 |
| Humidity may | 11 | 1.08 | 0.30 | 0.09 |
| Leaf C/N ratios | NA | NA | NA | NA |
| Dry weight | NA | NA | NA | NA |

Table S5.d Effect of environmental parameters in 2006 on the density of gall parasitoids in simple regression analysis (see Appendix 10). Note that for technical reasons humidity in March was not recorded on one tree. Thus sample size was not 18 but 17 trees.

|  | Effect on gall-parasitoid density | | | |
| --- | --- | --- | --- | --- |
|  | Df | T | p | R² |
| Crown size | 15 | -0.39 | 0.70 | 0.01 |
| Surrounding canopy density | 15 | -1.94 | 0.07 | 0.20 |
| Temperature march | 15 | -0.32 | 0.74 | 8*10^-3^ |
| Humidity march | 14 | -1.93 | 0.07 | 0.22 |
| Temperature april | 15 | -0.30 | 0.76 | 6*10^-3^ |
| Humidity april | 15 | -0.21 | 0.21 | 0.09 |
| Temperature may | 15 | 0.06 | 0.94 | 3*10^-4^ |
| Humidity may | 15 | -1.47 | 0.16 | 0.12 |
| Leaf C/N ratios | 15 | 0.76 | 0.45 | 0.03 |
| Dry weight | 15 | -0.08 | 0.93 | 5*10^-4^ |

Table S5.e Effect of environmental parameters on insect herbivory in 2010 in 2011 and in 2006, based on simple regression analyses.

|  | 2010 | | | | 2011 | | | |
| --- | --- | --- | --- | --- | --- | --- | --- | --- |
|  | Df | T | P | R² | Df | T | P | R² |
| crown size | 20 | -1.04 | 0.31 | 0.05 | 20 | -0.83 | 0.41 | 0.03 |
| crown volume | 20 | 1.22 | 0.23 | 0.06 | 20 | 0.76 | 0.45 | 0.02 |
| Temperature march | 19 | 0.62 | 0.53 | 0.02 | 13 | 3.06 | 8*10^-3^ | 0.42 |
| Humidity march | 19 | 0.15 | 0.87 | 1*10^-3^ | 13 | -0.94 | 0.36 | 0.06 |
| Temperature april | 19 | 1.18 | 0.24 | 0.06 | 13 | 2.54 | 0.02 | 0.33 |
| Humidity april | 19 | -0.11 | 0.90 | 7*10^-4^ | 13 | -1.12 | 0.27 | 0.08 |
| Temperature may | 18 | 0.29 | 0.77 | 4*10^-3^ | 13 | 2.40 | 0.03 | 0.30 |
| Humidity may | 20 | 1,08 | 0,29 | 0.05 | 13 | -0,97 | 0,34 | 0.06 |
| leaf C/N ratios | 20 | -0,64 | 0,52 | 0.02 | NA | NA | NA | NA |
| dry weight | 20 | -1,12 | 0,27 | 0.05 | NA | NA | NA | NA |

|  | 2006 | | | |
| --- | --- | --- | --- | --- |
|  | Df | T | P | R² |
| Crown size | 16 | 0.61 | 0.54 | 0.02 |
| Surrounding canopy density | 16 | -1.47 | 0.15 | 0.12 |
| Temperature march | 14 | 2.83 | 0.01 | 0.36 |
| Humidity march | 14 | -1.15 | 0.26 | 0.08 |
| Temperature april | 16 | 2.47 | 0.02 | 0.27 |
| Humidity april | 16 | -1.53 | 0.14 | 0.12 |
| Temperature may | 16 | 0.28 | 0.78 | 5*10^-3^ |
| Humidity may | 16 | -0.91 | 0.37 | 0.05 |
| Leaf C/N ratios | 16 | 1.16 | 0.25 | 0.07 |
| Dry weight | 16 | 0.43 | 0.66 | 0.01 |

Table S5.f Effect of environmental parameters on the budburst phenology in 2010 and in 2011 in simple regression analyses.

|  | Effect in 2010 | | | | Effect in 2011 | | | |
| --- | --- | --- | --- | --- | --- | --- | --- | --- |
|  | Df | T | P | R² | Df | T | P | R² |
| crown size | 20 | -0.27 | 0.78 | 3*10^-3^ | 20 | 0.10 | 0.91 | 5*10^-4^ |
| crown volume | 20 | 0.35 | 0.72 | 6*10^-3^ | 20 | 1.75 | 0.09 | 0.13 |
| Temperature march | 19 | -0.54 | 0.59 | 0.01 | 13 | -1.98 | 0.06 | 0.23 |
| Humidity march | 19 | 0.28 | 0.77 | 4*10^-3^ | 13 | 0.76 | 0.45 | 0.04 |
| Temperature april | 19 | -0.10 | 0.91 | 6*10^-4^ | 13 | -2.04 | 0.06 | 0.24 |
| Humidity april | 19 | 0.007 | 0.99 | 3*10^-6^ | 13 | 0.76 | 0.45 | 0.04 |
| Temperature may | 18 | 1.25 | 0.22 | 0.08 | 13 | -0.55 | 0.58 | 0.02 |
| Humidity may | 20 | -0.94 | 0.35 | 0.04 | 13 | 0.63 | 0.53 | 0.02 |

Table S5.g Effect of environmental parameters on ectophagous Lepidoptera density in 2010 and in 2011 in simple regression analyses.

|  | Effect in 2010 | | | | Effect in 2011 | | | |
| --- | --- | --- | --- | --- | --- | --- | --- | --- |
|  | Df | T | P | R² | Df | T | P | R² |
| crown size | 20 | -0.26 | 0.79 | 3*10^-3^ | 20 | -0.15 | 0.87 | 1*10^-3^ |
| crown volume | 20 | 0.05 | 0.95 | 1*10^-4^ | 20 | -1.71 | 0.10 | 0.12 |
| Temperature march | 19 | 0.79 | 0.43 | 0.03 | 13 | 2.15 | 0.05 | 0.26 |
| Humidity march | 19 | -1.54 | 0.13 | 0.11 | 13 | -0.70 | 0.49 | 0.03 |
| Temperature april | 19 | 0.80 | 0.43 | 0.03 | 13 | 3.28 | 0.005 | 0.45 |
| Humidity april | 19 | -1.38 | 0.18 | 0.09 | 13 | -0.84 | 0.41 | 0.05 |
| Temperature may | 18 | -0.47 | 0.64 | 0.01 | 13 | 2.11 | 0.05 | 0.25 |
| Humidity may | 20 | 0.78 | 0.43 | 0.03 | 13 | -0.66 | 0.51 | 0.03 |
| leaf C/N ratios | 20 | 0.95 | 0.34 | 0.04 | NA | NA | NA | NA |
| dry weight | 20 | -0.25 | 0.80 | 3*10^-3^ | NA | NA | NA | NA |

Table S5.h Effect of phylogenetic isolation of focal trees on environmental parameters in 2010 in 2011 and in 2006, based on simple regression analyses.

|  | 2010 | | | | 2011 | | | |
| --- | --- | --- | --- | --- | --- | --- | --- | --- |
| Relationship between phylogenetic isolation and | Df | T | P | R² | Df | T | P | R² |
| crown size | 20 | 1.52 | 0.14 | 0.10 | 20 | 1.52 | 0.14 | 0.10 |
| crown volume | 20 | -0.36 | 0.71 | 6*10^-3^ | 20 | -0.36 | 0.71 | 6*10^-3^ |
| Temperature march | 19 | -0.77 | 0.44 | 0.03 | 13 | -1.38 | 0.18 | 0.12 |
| Humidity march | 19 | 0.33 | 0.74 | 5*10^-3^ | 13 | 1.46 | 0.16 | 0.14 |
| Temperature april | 19 | -1.25 | 0.22 | 0.07 | 13 | -2.18 | 0.04 | 0.26 |
| Humidity april | 19 | 0.40 | 0.68 | 8*10^-3^ | 13 | 1.68 | 0.11 | 0.17 |
| Temperature may | 18 | -0.64 | 0.52 | 0.02 | 13 | -0.73 | 0.47 | 0.04 |
| Humidity may | 20 | -0.51 | 0.61 | 0.01 | 13 | 1.40 | 0.18 | 0.13 |
| leaf C/N ratios | 20 | -0.19 | 0.84 | 1*10^-3^ | NA | NA | NA | NA |
| dry weight | 20 | 0.51 | 0.61 | 0.01 | NA | NA | NA | NA |

|  | 2006 | | | |
| --- | --- | --- | --- | --- |
|  | Df | T | P | R² |
| Crown size | 16 | 0.09 | 0.92 | 5*10^-4^ |
| Surrounding canopy density | 16 | 3.06 | 7*10^-3^ | 0.36 |
| Temperature march | 14 | -0.73 | 0.47 | 0.03 |
| Humidity march | 14 | 1.38 | 0.18 | 0.12 |
| Temperature april | 16 | -1.30 | 0.21 | 0.09 |
| Humidity april | 16 | 1.39 | 0.18 | 0.10 |
| Temperature may | 16 | -0.07 | 0.93 | 3*10^-4^ |
| Humidity may | 16 | 1.24 | 0.23 | 0.08 |
| Leaf C/N ratios | 16 | -1.78 | 0.09 | 0.16 |
| Dry weight | 16 | -0.92 | 0.36 | 0.05 |

**Online Appendix S6. Species composition of ectophagous Lepidoptera parasitoids in 2010 and 2011.**

Table S6. The identification numbers from 1 to 250 correspond to the parasitoids of ectophagous Lepidoptera in 2010 and the identification numbers from 300 to 540 correspond to the parasitoids of ectophagous Lepidoptera in 2011. In the table Brac.: Braconidae; Ichn.: Ichneumonidae; Eul.: Eulophidae; Geo.: Geometridae; Las. : Lasiocampidae; Noc.: Noctuidae; Oec.: Oecophoridae; Pyr.: Pyralidae; Tor.: Tortricidae; Yps.: Ypsolophidae)

| Parasitoid species | Family, Sub-family | N individuals | Year of collection | Identified lepidopteran hosts |
| --- | --- | --- | --- | --- |
| *Aleiodes circumcriptus* | Brac., Rogadinae | 3 | 2010 | *Conistra erythrocephala* or *Eupsilia* sp. (Noc.) |
| *Aleiodes* sp1 | Brac. Rogadinae | 4 | 2011 | *Orthosia cerasi* (Noc.) |
| *Apanteles lineipes* | Brac., Microgastrinae | 1 | 2011 | *Zeiraphera isertana* (Tor.) |
| *Apanteles* sp1 | Brac., Microgastrinae | 3 | 2010 (1), 2011 (2) | Tortricidae spp. |
| *Apanteles* sp2 | Brac., Microgastrinae | 2 | 2010 | *Conistra erythrocephala* or *Eupsilia* sp. (Noc.), *Acrobasis repandana* (Pyr.) |
| *Apanteles* sp3 | Brac., Microgastrinae | 1 | 2010 | *Ypsolopha parenthesella* (Yps.) |
| *Apanteles* sp4 | Brac., Microgastrinae | 1 | 2010 | *Tortrix viridana* (Tor.) |
| *Apechtis quadridentata* | Ichn., Pimplinae | 1 | 2010 | *Orthosia cerasi* (Noc.) |
| *Apophua bipunctoria* | Ichn., Banchinae | 2 | 2010, 2011 | Tortricidae spp. |
| *Bassus rufipes* | Brac., Agathidinae | 2 | 2010, 2011 | *Hedya nubiferana* (Tor.), Tortricidae sp. |
| *Bassus dimidiator* | Brac., Agathidinae | 1 | 2011 | *Hedya nubiferana* (Tor.) |
| *Campoplex* sp1 | Ichn., Campopleginae | 1 | 2010 | *Ypsolopha parenthesella* (Yps.) |
| *Charmon cruentatus* | Brac., Charmontinae | 1 | 2011 | *Tortrix viridana* (Tor.) |
| *Cotesia spuria* | Brac., Microgastrinae | 1 | 2011 | *Poecilocampa populi* (Las.) |
| *Diadegma* sp1 | Ichn., Campopleginae | 8 | 2010 (6), 2011 (2) | *Agriopis aurantaria* (Geo.), *Archips* spp. (Tor.), Tortricidae spp. |
| *Exochus citripes* | Ichn., Metopiinae | 3 | 2010 | *Archips* spp. (Tor.) |
| *Exochus semilividus* | Ichn., Metopiinae | 1 | 2010 | *Archips* sp. (Tor.) |
| *Hyposoter* sp1 | Ichn., Campopleginae | 1 | 2011 | *Carcina quercana* (Oec.) |
| *Itoplectis alternans* | Ichn., Pimplinae | 1 | 2011 | ? |
| *Lissonota* sp. 1 | Ichn., Banchinae | 1 | 2010 | ? |
| *Macrocentrus bicolor* | Brac., Macrocentrinae | 20 | 2010 (9), 2011 (11) | *Archips* spp. (Tor.), *Carcina quercana* (Oec.), *Hedya nubiferana* (Tor.), *Tortrix viridana* (Tor.) |
| *Macrocentrus thoracicus* | Brac., Macrocentrinae | 1 | 2011 | *Hedya nubiferana* (Tor.) |
| *Mesochorus* sp1 | Ichn., Mesochorinae | 2 | 2010 | *Archips* sp. (Tor.), Tortricidae sp. |
| *Meteorus* sp1 | Brac., Euphorinae | 2 | 2010, 2011 | hyperparasitoid of *Hedya nubiferana* (Tor.) and *Acrobasis consociella* (Pyr.) |
| *Phytodietus polyzonias* | Ichn., Tryphoninae | 3 | 2010 | *Tortrix viridana* (Tor.) |
| *Scambus* sp1 | Ichn., Pimplinae | 1 | 2011 | ? |
| *Scambus* sp2 | Ichn., Pimplinae | 1 | 2010 | Geometridae sp. |
| *Sinophorus* sp1 | Ichn., Campopleginae | 1 | 2010 | *Conistra erythrocephala* (Noc.), |
| *Sinophorus* sp2 | Ichn., Campopleginae | 2 | 2011 | *Archips* sp. (Tor.), *Tortrix viridana* (Tor.) |
| *Sympiesis sericeicornis* | Eul., Eulophinae | 2 | 2011 | *Tortrix viridana* (Tor.) |
| *Sympiesis* sp1 | Eul., Eulophinae | 2 | 2010, 2011 | *Tortrix viridana* (Tor.) |
| *Sympiesis* sp2 | Eul., Eulophinae | 1 | 2011 | *Tortrix viridana* (Tor.) |
| *Sympiesis* sp3 | Eul., Eulophinae | 5 | 2010 (1), 2011 (4) | *Tortrix viridana* (Tor.), Tortricidae spp. |
| *Tetrastichus* sp1 | Eul., Tetrastichinae | 1 | 2011 | *Carcina quercana* (Oec.) |
| *Tranosema* sp1 | Ichn., Campopleginae | 2 | 2010, 2011 | *Agriopis aurantaria* (Geo.), *Tortrix viridana* (Tor.) |
| Larvae spp | Ichneumonidae? | 3 | 2011 | *Archips* spp. (Tor.), *Carcina quercana* (Oec.), *Conistra* sp. (Noc.) |
| Larvae spp | Ichneumonidae or Braconidae | 9 | 2010 (1), 2011 (8) | *Archips* spp. (Tor.), *Pammene argyrana* (Tor.), *Tortrix viridana* (Tor.) |

**Online Appendix S.7** **Variables correlated to species richness of ectophagous Lepidoptera parasitoids in 2010 and 2011**

Table S7 a. Simple and multiple regression analyses testing the effect of phylogenetic isolation, insect-herbivore density and insect herbivory on species richness of ectophagous Lepidoptera parasitoids in 2010.

| Model | Variable | Df | T | p |
| --- | --- | --- | --- | --- |
| R²=0.25 | Phylogenetic isolation | 14 | -2.21 | 0.04 |
| R²=0.45 | Ectophagous Lepidoptera density | 14 | 3.43 | 4*10^-3^ |
| R²=0.09 | Insect herbivory | 14 | 1.22 | 0.24 |
| R²=0.08 | Budburst phenology | 14 | -1.12 | 0.27 |
| R²=0.45  P=0.01 | Phylogenetic isolation  Ectophagous Lepidoptera density | 13  13 | -0.20  2.19 | 0.83  0.04 |
| R²=0.13  P=0.13 | Phylogenetic isolation  Insect herbivory | 13  13 | -1.74  -0.40 | 0.10  0.68 |
| R²=0.31  P=0.08 | Phylogenetic isolation  Budburst phenology | 13  13 | -2.11  -1.06 | 0.05  0.30 |

Table S7 b. Results of simple and multiple regression analyses testing the effect of phylogenetic isolation, insect-herbivore density and insect herbivory on species richness of ectophagous Lepidoptera parasitoids in 2011.

| Model | Variable | Df | T | p |
| --- | --- | --- | --- | --- |
| R²=0.06 | Phylogenetic isolation of host plant | 17 | -1.06 | 0.30 |
| R²=0.57 | Ectophagous Lepidoptera density | 17 | 4.84 | 1*10^-4^ |
| R²=0.01 | Insect herbivory | 17 | 0.44 | 0.66 |
| R²=0.10 | Budburst phenology | 17 | -1.41 | 0.17 |
| R²=0.58  P=9*10^-4^ | Phylogenetic isolation  Ectophagous Lepidoptera density | 16  16 | 0.36  4.46 | 0.71  0.04 |
| R²=0.06  P=0.59 | Phylogenetic isolation  Insect herbivory | 16  16 | -0.93  3*10^-3^ | 0.36  0.99 |
| R²=0.11  P=0.37 | Phylogenetic isolation  Budburst phenology | 16  16 | -0.42  -0.97 | 0.67  0.34 |

Conclusions for 2010 and 2011:

Phylogenetic isolation significantly decreases species richness of ectophagous Lepidoptera parasitoids only in 2010 while decreasing ectophagous Lepidoptera density decreases the species richness of ectophagous Lepidoptera parasitoids in 2010 and 2011. In all multivariate analyses, only ectophagous Lepidoptera density had a significant effect on species richness of ectophagous Lepidoptera parasitoids.

**Online Appendix 8**  **Model selection identifying variables directly affecting enemy pressure on ectophagous Lepidoptera (parasitism rate in 2010 and 2011, and bird predation in 2011)**

The goal of these analyses was to identify whether phylogenetic isolation remains a significant predictor of enemy pressure under different combinations of independent variables and hence whether phylogenetic isolation acts proximately on enemy pressure.

Table S8.1 Best regression models to predict parasitism rate in 2010 based on corrected Akaike I Criterion AICc. Models are ranked from the best to the worst model according to AICc. Tolerances of a given variable are one minus the R² of the relationship between the given independent variable with all other independent variables. Simple regression model with the best AICc is indicated but see also Table 1 for all simple regression analysis.

| Model | Variable | Df | T | p | Standardized regression coefficient | Tolerance |
| --- | --- | --- | --- | --- | --- | --- |
| r²=0.62  AIC= -40.95  AICc= -38.74 | Ectophagous Lepidoptera density | 19 | 5.62 | 2*10^-5^ |  |  |
| p=8*10^-5^  r²=0.64  AIC= -40.35  AICc= -35.68 | Insect herbivory  Ectophagous Lepidoptera density | 18  18 | -1.11  5.08 | 0.27  7*10^-5^ | -0.20  0.91 | 0.59  0.59 |
| p=1*10^-4^  r²=0.63  AIC= -39.71  AICc= -35.04 | Ectophagous Lepidoptera density  Bud burst phenology | 18  18 | 3.66  -0.81 | 1*10^-3^  0.42 | 0.68  -0.15 | 0.56  0.56 |
| p=1*10^-4^  r²=0.62  AIC= -38.96  AICc= -34.29 | Phylogenetic isolation  Ectophagous Lepidoptera density | 18  18 | -0.10  3.35 | 0.91  3*10^-3^ | -0.02  0.77 | 0.39  0.39 |
| p=3*10^-4^  r²=0.65  AIC= -38.67  AICc= -31.26 | Ectophagous Lepidoptera density  Insect herbivory  Bud burst phenology | 18  18  18 | 3.35  -0.89  -0.51 | 3*10^-3^  0.38  0.61 | 0.83  -0.17  -0.10 | 0.32  0.54  0.51 |
| p=3*10^-4^  r²=0.65  AIC= -38.56  AICc= -31.14 | Phylogenetic isolation  Ectophagous Lepidoptera density  Insect herbivory | 17  17  17 | -0.41  3.58  -1.16 | 0.68  2*10^-3^  0.26 | -0.09  0.85  -0.22 | 0.36  0.35  0.55 |
| p=4*10^-4^  r²=0.63  AIC= -37.79  AICc= -30.37 | Phylogenetic isolation  Ectophagous Lepidoptera density  Bud burst phenology | 17  17  17 | -0.24  2.25  -0.82 | 0.80  0.03  0.41 | -0.05  0.63  -0.16 | 0.38  0.26  0.55 |
| p=1*10^-3^  r²=0.53  AIC= -34.27  AICc= -29.61 | Phylogenetic isolation  Bud burst phenology | 18  18 | -2.50  -2.32 | 0.02  0.03 | -0.44  -0.41 | 0.81  0.81 |
| p=1*10^-3^  r²=0.65  AIC= -36.95  AICc= -26.45 | Phylogenetic isolation  Ectophagous Lepidoptera density  Insect herbivory  Budburst phenology | 16  16  16  16 | -0.46  2.44  -0.95  -0.54 | 0.64  0.02  0.35  0.59 | -0.11  0.75  -0.19  -0.11 | 0.36  0.22  0.51  0.51 |
| p=4*10^-3^  r²=0.53  AIC= -32.27  AICc= -24.86 | Phylogenetic isolation  Insect herbivory  Bud burst phenology | 17  17  17 | -1.95  -3*10^-3^  -2.26 | 0.06  0.99  0.03 | -0.44  -7*10^-4^  -0.41 | 0.52  0.60  0.81 |
| p=0.01  r²=0.39  AIC= -28.75  AICc= -24.09 | Phylogenetic isolation  Insect herbivory | 18  18 | -2.69  -0.07 | 0.01  0.94 | -0.63  -0.01 | 0.60  0.60 |

Table S8.2 Best regression models to predict parasitism rate of exophagous Lepidoptera in 2011 based on Akaike I Criterion. Models are ranked from the best to the worst model according to AICc. Tolerances of a given variable are one minus R² of the relationship between the given independent variable with all other independent variables. Simple regression model with the best AICc are indicated but see also Table 2 for all simple regression analysis.

| Model | Variable | Df | T | p | Standardized regression coefficient | Tolerance |
| --- | --- | --- | --- | --- | --- | --- |
| r²=0.22  AIC= -17.75  AICc= -15.55 | Phylogenetic isolation | 20 | -2.38 | 0.02 |  |  |
| r²=0.17  AIC= -16.58  AICc= -14.38 | Ectophagous Lepidoptera density | 20 | 2.08 | 0.05 |  |  |
| p=0.05  r²=0.26  AIC= -16.99  AICc= -12.36 | Phylogenetic isolation  Bud burst phenology | 19  19 | -1.62  -1.05 | 0.12  0.30 | -0.36  -0.23 | 0.78  0.78 |
| p=0.055  r²=0.26  AIC= -16.94  AICc= -12.30 | Phylogenetic isolation  Ectophagous Lepidoptera density | 19  19 | -1.46  1.02 | 0.15  0.31 | -0.34  0.23 | 0.71  0.71 |
| p=0.06  r²=0.25  AIC= -16.76  AICc= -12.13 | Ectophagous Lepidoptera density  Bud burst phenology | 19  19 | 1.55  -1.40 | 0.13  0.17 | 0.32  -0.29 | 0.89  0.89 |
| p=0.06  r²=0.25  AIC= -16.58  AICc= -11.95 | Insect herbivory  Ectophagous Lepidoptera density | 19  19 | 1.34  1.92 | 0.19  0.06 | 0.26  0.38 | 0.98  0.98 |
| p=0.08  r²=0.23  AIC= -16.01  AICc= -11.38 | Phylogenetic isolation  Insect herbivory | 19  19 | -1.76  0.47 | 0.09  0.63 | -0.41  0.11 | 0.73  0.73 |
| p=0.08  r²=0.29  AIC= -16.00  AICc= -8.66 | Phylogenetic isolation  Ectophagous Lepidoptera density  Bud burst phenology | 18  18  18 | -1.02  0.91  -0.94 | 0.32  0.37  0.35 | -0.25  0.21  -0.21 | 0.61  0.70  0.77 |
| p=0.08  r²=0.29  AIC= -15.99  AICc= -8.65 | Ectophagous Lepidoptera density  Insect herbivory  Bud burst phenology | 18  18  18 | 1.50  1.01  -1.08 | 0.14  0.32  0.29 | 0.31  0.21  -0.23 | 0.88  0.91  0.82 |
| p=0.10  r²=0.28  AIC= -15.50  AICc= -8.16 | Phylogenetic isolation  Ectophagous Lepidoptera density  Insect herbivory | 18  18  18 | -0.87  1.12  0.68 | 0.39  0.27  0.50 | -0.24  0.27  0.16 | 0.51  0.68  0.71 |
| p=0.12  r²=0.27  AIC= -15.20  AICc= -7.86 | Phylogenetic isolation  Insect herbivory  Bud burst phenology | 18  18  18 | -1.24  0.40  -0.99 | 0.22  0.68  0.33 | -0.31  0.09  -0.22 | 0.62  0.73  0.77 |
| p=0.15  r²=0.31  AIC= -14.45  AICc= -4.09 | Phylogenetic isolation  Ectophagous Lepidoptera density  Insect herbivory  Budburst phenology | 17  17  17  17 | -0.59  0.99  0.59  -0.86 | 0.55  0.33  0.56  0.39 | -0.17  0.24  0.14  -0.20 | 0.47  0.67  0.70  0.76 |

Table S8.3 Best regression models to predict bird predation on exophagous Lepidoptera in 2011. Tolerance indicates the proportion of the variance of a given independent variable not explained by the other independent variables in a multiple regression model. Tolerances of a given variable are one minus the R² of the relationship between the given independent variable with all other independent variables. See Table 3 for simple regression analysis. Note that AIC and AICc were not included as phylogenetic isolation has never a significant effect on bird predation.

| Model | Variable | Df | T | p | Standardized regression coefficient | Tolerance |
| --- | --- | --- | --- | --- | --- | --- |
| p=0.70  r²=0.12 | Phylogenetic isolation  Ectophagous Lepidoptera density  Insect herbivory  Budburst phenology | 15  15  15  15 | 0.59  0.53  -0.42  -1.19 | 0.56  0.59  0.67  0.25 | 0.20  0.16  -0.11  -0.33 | 0.49  0.62  0.74  0.72 |
| p=0.68  r²=0.08 | Phylogenetic isolation  Ectophagous Lepidoptera density  Bud burst phenology | 16  16  16 | 0.59  0.65  -0.86 | 0.55  0.52  0.39 | 0.18  0.19  -0.24 | 0.58  0.62  0.72 |
| p=0.68  r²=0.04 | Phylogenetic isolation  Ectophagous Lepidoptera density | 17  17 | 0.33  0.84 | 0.74  0.40 | 0.09  0.24 | 0.65  0.65 |
| p=0.56  r²=0.06 | Ectophagous Lepidoptera density  Bud burst phenology | 17  17 | 0.42  -0.72 | 0.67  0.47 | 0.11  -0.18 | 0.81  0.81 |
| p=0.58  r²=0.06 | Phylogenetic isolation  Bud burst phenology | 17  17 | 0.32  -1.03 | 0.74  0.31 | 0.08  -0.27 | 0.75  0.75 |

**Online Appendix S9. Model selection identifying variables controlling variables directly affecting parasitism rate, i.e. ectophagous Lepidoptera density, insect herbivory, and budburst phenology.**

Dependent variables considered are ectophagous Lepidoptera density, insect herbivory, and budburst phenology. Out of these ectophagous Lepidoptera density is the prime proximate control of the parasitism rate of these Lepidoptera. Independent variables considered are phylogenetic isolation of focal trees, ectophagous Lepidoptera density, insect herbivory, budburst phenology and the physico-chemical characteristics that scored significant in the corresponding simple regression analyses in Appendix 5.

Table S9.1 Best regression models to predict insect herbivory (a), ectophagous Lepidoptera density, (b) and budburst phenology (c) in 2010 and in 2011 based on Akaike I Criterion. Models are ranked from the best to the worst model according to AICc. Note that in 2011, for technical reasons Temperature and humidity was not recorded on 7 trees. Thus, in models including temperature or humidity in 2011, sample size was not 22 but 15 trees.

| Model | Variable | | Df | T | p | Standardized regression coefficient | Tolerance |
| --- | --- | --- | --- | --- | --- | --- | --- |
| 1. Predicting insect herbivory in 2010 | | | | | | | |
| r²=0.42  AIC= -85.12  AICc= -82.92 | Ectophagous Lepidoptera density | | 20 | 3.80 | 1 *10^-3^ | 0.64 | NA |
| r²=0.41  AIC= -84.73  AICc= -82.53 | Phylogenetic isolation | | 20 | -3.72 | 1 *10^-3^ | -0.64 | NA |
| p=2*10^-3^  r²=0.46  AIC= -84.85  AICc= -80.22 | Phylogenetic isolation  Ectophagous Lepidoptera density | | 19  19 | -1.24  1.38 | 0.22  0.18 | -0.34  0.37 | 0.37  0.37 |
| p=2*10^-3^  r²=0.46  AIC= -84.82  AICc= -80.19 | Ectophagous Lepidoptera density  Budburst phenology | | 19  19 | 3.79  1.23 | 1 *10^-3^  0.23 | 0.81  0.26 | 0.61  0.61 |
| p=6*10^-3^  r²=0.41  AIC= -82.77  AICc= -78.14 | Phylogenetic isolation  Budburst phenology | | 19  19 | -3.38  0.17 | 3 *10^-3^  0.86 | -0.65  0.03 | 0.82  0.82 |
| p=5*10^-3^  r²=0.49  AIC= -84.20  AICc= -76.87 | Phylogenetic isolation  Budburst phenology  Ectophagous Lepidoptera density | | 18  18  18 | -1.07  1.06  1.74 | 0.29  0.30  0.09 | -0.29  0.22  0.55 | 0.36  0.60  0.27 |
| r²=0.05  AIC= -74.39  AICc= -72.39 | Budburst phenology | | 20 | -1,08 | 0,28 | -0.23 | NA |
| (a) Predicting insect herbivory in 2011 | | | | |  |  |  |
| r²=0.26  AIC= -86.88  AICc= -84.6 | Phylogenetic isolation | | 20 | -2.66 | 0.01 | -0.51 | NA |
| p=0.04  r²=0.28  AIC= -85.66  AICc= -81.03 | Phylogenetic isolation  Ectophagous Lepidoptera density | | 19  19 | -2.67  -0.82 | 0.01  0.41 | -0.61  -0.19 | 0.71  0.71 |
| p=0.05  r²=0.26  AIC= -84.99  AICc= -80.36 | Phylogenetic isolation  Budburst phenology | | 19  19 | -2.15  -0.31 | 0.04  0.76 | -0.47  -0.06 | 0.78  0.78 |
| r²=0.08  AIC= -82.17  AICc= -79.97 | Budburst phenology | | 20 | -1,36 | 0,18 | -0.29 | NA |
| r²=0.01  AIC= -80.63  AICc= -78.43 | Ectophagous Lepidoptera density | | 20 | 0.62 | 0.53 | 0.13 | NA |
| r²=0.42  AIC= -63.47  AICc= -61.17 | Temperature (March) | | 13 | 3,06 | 8*10^-3^ | 0.64 | NA |
| p=0.03  r²=0.43  AIC= -61.83  AICc= -56.83 | Phylogenetic isolation  Temperature (March) | | 12  12 | -0.53  2.59 | 0.60  0.02 | -0.12  0.60 | 0.87  0.87 |
| r²=0.33  AIC= -61.36  AICc= -59.06 | Temperature (April) | | 13 | 2,54 | 0,02 | 0.57 | NA |
| r²= 0.30  AIC= -60.83  AICc= -58.53 | Temperature (May) | | 13 | 2,40 | 0,03 | 0.55 | NA |
| p=0.06  r²=0.36  AIC= -60.08  AICc= -55.08 | Phylogenetic isolation  Temperature (May) | | 12  12 | -1.01  2.15 | 0.32  0.05 | -0.23  0.50 | 0.95  0.95 |
| p=0.08  r²=0.33  AIC= -59.41  AICc= -54.41 | Phylogenetic isolation  Temperature (April) | | 12  12 | -0.21  1.98 | 0.83  0.07 | -0.05  0.54 | 0.73  0.73 |
| 1. Predicting ectophagous Lepidoptera density in 2010 | | | | | | | |
| r²=0.72  p=4*10^-6^  AIC= - 82.10  AICc= -77.47 | Phylogenetic isolation  Budburst phenology | | 19  19 | -4,87  -2,64 | 1 * 10^-4^  0,01 | -0.64  -0.34 | 0.82  0.82 |
| r²=0.62  AIC= -77.20  AICc= -75 | Phylogenetic isolation | | 20 | -5.74 | 1 * 10^-5^ | -0.78 | NA |
| r²=0.38  AIC= -66.27  AICc= -64.07 | Budburst phenology | | 20 | -3,50 | 2 * 10^-3^ | -0.61 | NA |
| (b) Predicting ectophagous Lepidoptera density in 2011 | | | | |  |  |  |
| r²=0.28  AIC= -91.30  AICc= -89.10 | Phylogenetic isolation | | 20 | -2.83 | 0.01 | -0.53 | NA |
| p=0.03  r²=0.29  AIC= -89.63  AICc= -85 | Phylogenetic isolation  Budburst phenology | | 19  19 | -2.24  -0.47 | 0.03  0.64 | -0.48  -0.10 | 0.78  0.78 |
| r²=0.10  AIC= -86.47  AICc= -84.27 | Budburst phenology | | 20 | -1,56 | 0,13 | -0.32 | NA |
| r²=0.45  AIC= -62.50  AICc= -60.20 | Temperature (April) | | 13 | 3.28 | 5*10^-3^ | 0.67 | NA |
| r²=0.49  p=0.01  AIC= -61.68  AICc= -56.68 | Phylogenetic isolation  Temperature (April) | | 12  12 | -0.98  2.29 | 0.34  0.04 | -0.23  0.55 | 0.73  0.73 |
| r²=0.26  AIC= -58.02  AICc= -55.72 | Temperature (March) | | 13 | 2.15 | 0.05 | 0.51 | NA |
| r²=0.25  AIC= -57.87  AICc= -55.57 | Temperature (May) | | 13 | 2.11 | 0.05 | 0.50 | NA |
| r²=0.44  p=0.03  AIC= -60.15  AICc= -55.15 | Phylogenetic isolation  Temperature (May) | | 12  12 | -1.99  1.90 | 0.06  0.08 | -0.43  0.41 | 0.95  0.95 |
| r²=0.39  p=0.04  AIC= -58.96  AICc= -53.96 | Phylogenetic isolation  Temperature (March) | | 12  12 | -1.61  1.55 | 0.13  0.14 | -0.38  0.37 | 0.87  0.87 |
| 1. Predicting budburst phenology in 2010 | | | | | | | |
|  | |  |  |  |  |  |  |
| r²=0.17  AIC= 124.58  AICc=126.78 | | Phylogenetic isolation | 20 | 2.03 | 0.05 | 0.41 | NA |
| (c) Predicting Budburst phenology in 2011 | | | | | | | |
| r²=0.24  AIC=76.47  AICc=78.77 | Temperature (April) | | 13 | -2.04 | 0.06 | -0.49 | NA |
| r²=0.23  AIC= 76.68  AICc=78.98 | Temperature (March) | | 13 | -1.98 | 0.06 | -0.48 | NA |
| p=0.09  r²=0.32  AIC= 76.73  AICc=81.73 | Phylogenetic isolation  Temperature (March) | | 12  12 | 1.29  -1.43 | 0.22  0.17 | 0.32  -0.36 | 0.87  0.87 |
| p=0.11  r²=0.29  AIC=77.31  AICc=82.31 | Phylogenetic isolation  Temperature (April) | | 12  12 | 0.98  -1.23 | 0.34  0.24 | 0.27  -0.34 | 0.73  0.73 |
| r²=0.21  AIC=118.01  AICc=120.1 | Phylogenetic isolation | | 20 | 2.34 | 0.02 | 0.46 | NA |

**Online Appendix S10. Effect of phylogenetic isolation of host tree on the density of galls and their parasitoids.**

We studied parasitoid species of highly specialist insect herbivores, oak galls produced by cynipid wasps (Hymenoptera: Cynipidae). We focused particularly on chalcidoid parasitoids which are known to be the primary cause of parasitoid-induced mortality on oak gall-insects (Hayward and Stone, 2005). This study was conducted in 2006.

**Information about the focal trees, phylogenetic isolation, insect herbivory.**

The forest of the 2006 study was the same as in 2010 and 2011. Eighteen c. 80-year-old focal trees (in 2006) with mean circumference ± SD at breast height = 93.2 ± 22.4 cm were used. As in 2010 and 2011, the trees were sampled in pairs (See Methods for details). We found that tree “pair” did not significantly affect gall parasitoid density in 2006 (ANOVA, d.f. =8, F =0.34, P =0.92), or gall density (d.f. =15 , F =0.67 , P =0.70). Thus we pooled tree pair with the error term. We also found that *Q. petraea* vs. *Q. robur* had no significant impact on gall parasitoid density (ANOVA, d.f. =15, F =0.12 , P =0.72) or on gall density (d.f. =15 , F =1.32 , P =0.26).

Phylogenetic isolation was calculated as in 2010 and 2011. Overall, phylogenetic isolation ranged from 5.71 to 106.67 million years. As in 2010 and 2011, diversity of the surrounding canopy, species richness of the surrounding canopy, distance to the closest conspecific oak and percentage of neighboring oaks were calculated (See methods for details).

Insect herbivory was measured like in 2010 and 2011. In 2006 mean percentages of leaf damage per tree ranged from 3 to 34%.

**Sampling design of parasitoids and their host galls.**

Chalcidoids were sampled monthly from late May to mid-November 2006, with flight interception traps (one per tree) placed in crowns at a mean height of 13 m above the ground (s.d. = 4.1 m). These traps are well established as a means of studying faunal composition of actively or passively flying insects in tree crowns (e.g. Goßner et al. 2009, Vialatte et al. 2010). We opted for flight interception traps because they permit an overview of the relatively long period of gall establishment and hence exposure to parasitoids. Chalcidoids were determined by Jean-Luc Vago in 2006. In total, 89 female (F) and 80 male (M) parasitoids of species that are known to be specialists of oak-galls were sampled, belonging to only 5 species: *Mesopolobus dubius* 87% of all F, 88% of all M*, Mesopolobus fuscipes,*  7% of all F, 11% of all M, *Mesopolobus tibialis,* 1% of all F, 0% of all M*, Arthrolytus occelus,* 1% of all F, 0% of all M, and *Sycophila variegata,* 1% of all F, 0% of all M. We only analyzed *Mesopolobus dubius* considering the very low abundance of the other species. In total, 78 females and 69 males of *Mesopolobus dubius* were sampled. In order to obtain the more accurate proxy of the density of truly active parasitoids, we only considered the females of this species. However, pooling both males and females did not change the conclusions of the analyses on phylogenetic isolation (See Table S10.1 and S10.2). One outlier exceeding all others values by 200 percent was excluded. Excluding this extreme observation did not change the sign of the relationship. Moreover non parametric analysis on the entire dataset led to the same conclusion (see below).

Galls were sampled in late August, early September 2006. Branches were sampled from each of the crowns, from the top stratum (the highest quarter of the tree, in proximity to the trunk), the lower but exposed stratum (in the outer canopy, with no branches above preventing sun exposure) and from a sheltered stratum (typically lower in the canopy, always with branches above reducing sun exposure). Galls were sampled directly from the leaves or buds by cutting between 1.5 and 2 m of branch. Overall, 269 insect galls were sampled on 23196 leaves. For all analyses, we pooled the samples from a given tree to calculate total gall density. The density of galls corresponded to the number of galls found on a branch divided by the number of leaves on this branch. To approach a potential parasitism rate, only cynipid gall species known to be parasitized by *Mesopolobus dubius* and to be specialist of oak were considered in further analysis (*Neuroterus quercusbaccarum, Neuroterus numismalis; Cynipini* ; see Stone et al. 2002 for their specialization on oak species). We use the information of host range of *Mesopolobus dubius* available on Universal Chalcidoidea database (2011). As indicated in the Universal Chalcidoidea database, one single record of *Mesopolobus dubius* indicates *Sparganothis pilleriana* (Tortricidae, Lepidoptera) as a host but a non-cynipid host record must be excluded as parasitoids of oak galls should be found only in the cynipids (Stone et al. 2002). Based on this information *Mesopolobus dubius* is a specialist of gall hosts only.

**Statistical analysis.**

Gall parasitoids and their hosts were analyzed separately. The statistical analysis performed was the same as the statistical analysis performed for enemy pressure of Lepidoptera (See Methods), i.e. simple regressions, multiple regressions to identify whether phylogenetic isolation has a proximate effect on galls or parasitoid abundances, multiple regressions to identify whether phylogenetic isolation controls proximate effects other than phylogenetic isolation and has an ultimate effect.

**Effect of surrounding canopy characteristics**

Tree species diversity of the surrounding canopy had no significant effect on gall parasitoid density (d.f.=15; t=0.15; P= 0.87), on gall density (d.f.=15; t=0.51; P= 0.61) or on herbivory (d.f.=16; t=-0.47; P= 0.63). Besides, tree species diversity of the surrounding canopy is not significantly related to phylogenetic isolation (d.f.=16; t=1.01; P=0.32).

Species richness of the surrounding canopy has no significant effect on gall parasitoid density (d.f.=15; t=0.83; P=0.41), on gall density (d.f.=15; t=0.85; P=0.40) or on herbivory (d.f.=16; t=-0.78; P=0.44). Besides, species richness of the surrounding canopy is not significantly related to phylogenetic isolation (d.f.=16; t=0.53; P=0.59).

Distance to the closest conspecific host tree had a marginally significant negative effect on gall parasitoid density (d.f.=15; t=-1.88; P= 0.07) but not on gall density (d.f.=15; t=1.68; P=0.11) or on herbivory (d.f.=16; t=-1.37; P= 0.18).

**Effect of phylogenetic isolation of focal trees**

Phylogenetic isolation was negatively correlated to insect herbivory in 2006 (d.f.=16; t=-3.62; p=2*10^-3^; r²=0.45; standardized regression coefficient or b* =-0.65). Female parasitoid density declined significantly with increasing phylogenetic isolation of the host plant and with decreasing insect herbivory (see Table S10.1 and Figure S10.2), while gall density increased significantly with phylogenetic isolation and decreased significantly with insect herbivory (See Table S10.3). These results indicates that female parasitoid density is very probably affected more by phylogenetic isolation and insect herbivory than by the gall density. In the multiple regression analyses, phylogenetic isolation is always significant and included in the best models to predict female-parasitoid density (See Table S10.1 and Table S10.1). This suggests that phylogenetic isolation has a proximate, direct, effect on female-parasitoid abundance. Moreover, phylogenetic isolation was always included in models explaining the other proximate variable to predict female parasitoid density (i.e. insect herboviry, see Table S10.4). This suggests that phylogenetic isolation also has an ultimate, indirect, effect on female-parasitoid abundance via insect herbivory (Fig. 10.2).

**Why phylogenetic isolation of host plants decreases density of gall parasitoids**

For gall parasitoids, just as for ectophagous Lepidoptera parasitoids, finding its host depends on dispersal and host seeking abilities. Parasitoids can disperse at a scale of kilometers (Santos et al. 2011), and thus, gall parasitoids might not be limited by their dispersal ability. However, distance to the focal oak had a marginally significant effect on parasitoid density and hence spatial isolation may additionally decrease parasitoid density, but the effect appears to be much less important than the effect of phylogenetic isolation. Phylogenetic isolation of the trees might negatively affect seeking of gall hosts both directly and indirectly. The direct effect may be the same as discussed for ectophagous Lepidoptera parasitoids: phylogenetically distantly related tree neighbors may act as repellent cues for parasitoids of oak galls and hide suitable gall hosts and their host trees. The indirect effect of phylogenetic isolation on gall parasitoid density might operate via a decline in host density. However, we found that gall parasitoid density did not correlate significantly with the density of galls in simple regression analysis. This suggests that galls emit or induce few or no cues that their parasitoids could seek across long distances (as already suggested by Tooker and De Moraes, 2007). Indeed, galling insects are known to provoke no serious damage to the host plant (in Mattson et al. 1988) and hence might not trigger emission of plant volatiles. Instead, surprisingly, density of gall parasitoids was correlated to insect herbivory caused by ectophagous insect herbivores. Thus, gall parasitoids may have to seek cues induced by damage resulting from non-galling ectophagous insect herbivores. In addition, oaks may not have specific cues corresponding to specific insect herbivores. Such an insect-species specificity of cues might be too costly for oaks given the large number of herbivore species feeding on oaks. The indirect effect of host-tree phylogenetic isolation on density of gall parasitoids is hence mediated by herbivory of ectophagous insect herbivores. As insect herbivory declines with phylogenetic isolation of host trees, gall parasitoids might face a severe problem in seeking the numerous gall hosts on these trees.

**Tables and Figures.**

**Table S10.1** Best regression models predicting female-parasitoid density from phylogenetic isolation, gall density and insect herbivory. Models are ranked from the best to the worst model according to AICc. Note that budburst phenology data were not available for 2006.

| Model | Variable | Df | T | p | Standardized regression coefficient |
| --- | --- | --- | --- | --- | --- |
| R²=0.43  AIC=80.36  AICc=82.62 | Phylogenetic isolation | 15 | -3.40 | 3*10^-3^ | -0.66 |
| R²=0.38  AIC=81.92  AICc=84.18 | Insect herbivory 2006 | 15 | 3.04 | 8*10^-3^ | 0.61 |
| R²=0.49  P=8*10^-3^  AIC=80.46  AICc= 85.31 | Phylogenetic isolation  Insect herbivory 2006 | 14  14 | -1.77  1.28 | 0.09  0.21 | -0.44  0.32 |
| R²=0.45  P=0.01  AIC=81.91  AICc=86.76 | Phylogenetic isolation  Gall density 2006 | 14  14 | -3.19  0.61 | 6*10^-3^  0.54 | -0.73  0.14 |
| R²=0.39  P=0.02  AIC=83.46  AICc=88.31 | Gall density 2006  Insect herbivory 2006 | 14  14 | 0.61  2.83 | 0.54  0.01 | 0.15  0.70 |
| R²=0.54  P=0.01  AIC=80.85  AICc=88.69 | Phylogenetic isolation  Insect herbivory 2006  Gall density 2006 | 13  13  13 | -2.01  1.59  1.13 | 0.06  0.13  0.27 | -0,51  0,42  0,26 |
| R²=0.05  AIC=89.19  AICc=91.45 | Gall density | 15 | -0.91 | 0.37 | -0.22 |

**Table S10.2** Best regression models predicting female plus male-parasitoid density from phylogenetic isolation, gall density and insect herbivory. Models are ranked from the best to the worst model according to AICc. Note that budburst phenology data were not available for 2006.

| Model | Variable | Df | T | p | Standardized regression coefficient |
| --- | --- | --- | --- | --- | --- |
| R²=0.49  AIC=103.94  AICc=106.30 | Phylogenetic isolation | 15 | -3.80 | 1*10^-3^ | -0.70 |
| R²=0.48  AIC=104.11  AICc=106.37 | Insect herbivory 2006 | 15 | 3.76 | 1*10^-3^ | 0.69 |
| R²=0.58  P=1*10^-3^  AIC=102.30  AICc=107.15 | Phylogenetic isolation  Insect herbivory 2006 | 14  14 | -1.87  1.82 | 0.08  0.08 | -0.42  0.41 |
| R²=0.44  P=0.04  AIC=101.88  AICc=109.72 | Phylogenetic isolation  Gall density 2006  Insect herbivory 2006 | 13  13  13 | -2.21  1.40  2.24 | 0,04  0,18  0,04 | -0,50  0,28  0.52 |
| R²=0.50  P=6*10^-3^  AIC=105.34  AICc=110.19 | Gall density 2006  Insect herbivory 2006 | 14  14 | 0.81  3.55 | 0,43  3*10^-3^ | 0.18  0.79 |
| R²=0.50  P=7*10^-3^  AIC=105.46  AICc=110.31 | Phylogenetic isolation  Gall density 2006 | 14  14 | -3.52  0.62 | 3*10^-3^  0,53 | -0.76  0.13 |
| R²=0.06  AIC=114.29  AICc=116.55 | Gall density | 15 | -1.01 | 0.32 | -0.25 |

**Table S10.3** Best regression models predicting gall density from phylogenetic isolation and insect herbivory. Models are ranked from the best to the worst model according to AICc. Note that budburst phenology data were not available for 2006..

| Model | Variable | Df | T | p | R² | Standardized regression coefficient |
| --- | --- | --- | --- | --- | --- | --- |
| AIC= -94.34  AICc= -92.09 | Insect herbivory 2006 | 16 | -2.40 | 0.02 | 0.26 | -0.51 |
| AIC= -93.96  AICc= -91.71 | Phylogenetic isolation | 16 | 2.31 | 0.03 | 0.25 | 0.50 |
| P= 0.37  AIC= -93.43  AICc= -88.63 | Phylogenetic isolation  Insect herbivory 2006 | 15  15 | 0.96  -1.13 | 0.34  0.27 | 0.30 | 0.27  -0.32 |

**Table S10.4** Best regression models predicting insect herbivory from phylogenetic isolation and environmental variables. Models are ranked from the best to the worst model according to AICc. Note that budburst phenology data were not available for 2006..

| Model | Variable | Df | T | p | Standardized regression coefficient | Tolerance |
| --- | --- | --- | --- | --- | --- | --- |
| r²= 0.45  AIC= -37.94  AICc= -35.69 | Phylogenetic isolation | 16 | -3.62 | 2*10^-3^ | -0.67 | NA |
| p=2*10^-3^  r²=0.56  AIC= -40.04  AICc= -35.24 | Phylogenetic isolation  Temperature (April) | 15  15 | -3,13  1.95 | 6*10^-3^  0.06 | -0.56  0.35 | 0.90  0.90 |
| p=1*10^-3^  r²=0.63  AIC= -37.43  AICc= -32.51 | Phylogenetic isolation  Temperature (March) | 13  13 | -3,13  2.94 | 7*10^-3^  0.01 | -0.53  0.50 | 0.96  0.96 |
| r²= 0.27  AIC= -32.95  AICc= -30.70 | Temperature (April) | 16 | 2.47 | 0.02 | 0.52 | NA |
| r²= 0.36  AIC= -30.41  AICc= -28.13 | Temperature (March) | 14 | 2.83 | 0.01 | 0.60 | NA |
| p=4*10^-3^  r²=0.64  AIC= -35.77  AICc= -27.77 | Phylogenetic isolation  Temperature (March)  Temperature (April) | 12  12  12 | -3.02  1.87  -0.50 | 0.01  0.08  0.62 | -0.56  0.65  -0.18 | 0.83  0.24  0.22 |

**Figure S10.1** Relationships between phylogenetic isolation of focal oak trees from neighboring trees and female-parasitoid density (a), and gall density (b). The statistics for these relationships are, respectively: (a) d.f. = 15, r² = 0.43, t = -3.40, P = 3*10^-3^ and (b) d.f. = 16, r² = 0.25, t = 2.31, P = 0.03.

**Figure S10.1**

(a)


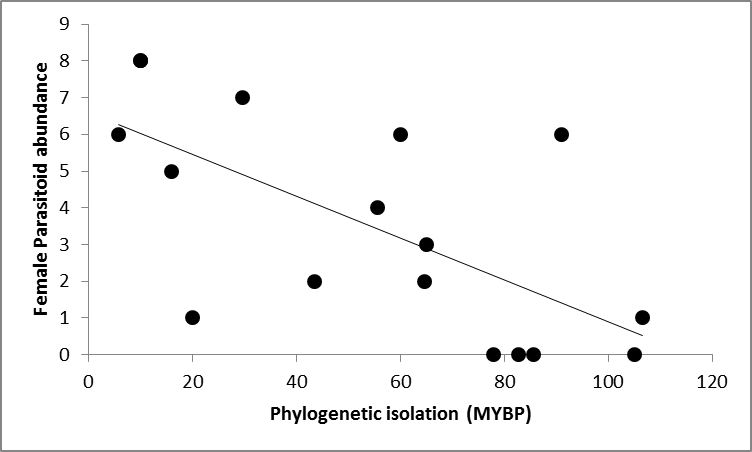


(b)


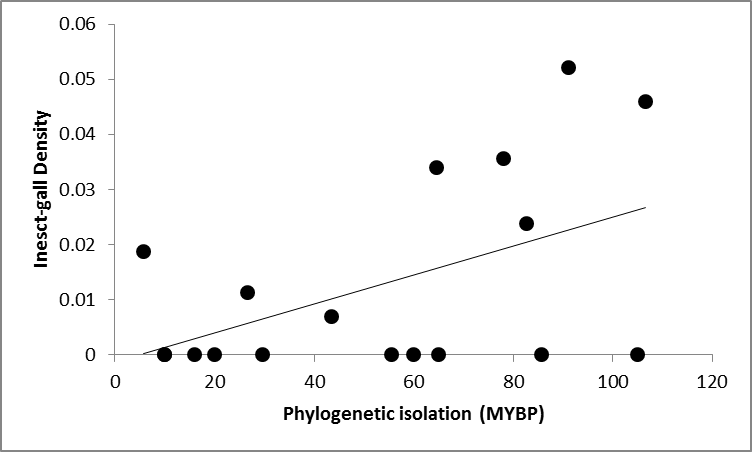


**Figure S10.2**

Figure representing the most probable link between variables that explain gall-parasitoid density based on the different analyses described in the Methods.

**
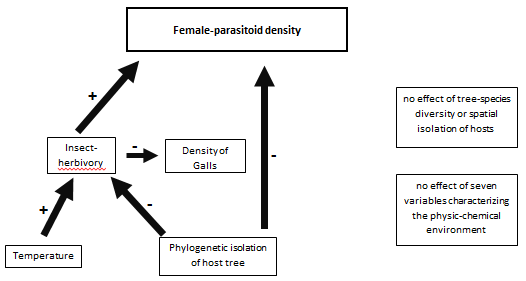
**

**Bibliography:**

Hayward A, Stone GN (2005). Oak Gall wasp communities: Evolution and ecology. Basic Appl Ecol 6: 435-443.

Mattson WJ (1980). Herbivory in relation with plant nitrogen-content. Annu Rev Ecol Syst 11: 119-161.

Noyes JS (2011) Universal Chalcidoidea Database. World Wide Web electronic publication. http://www.nhm.ac.uk/chalcidoids

Stone GN, Schonrogge K, Atkinson RJ, Bellido D & Pujade-Villar J (2002) The population biology of oak gall wasps (Hymenoptera : Cynipidae). Annu Rev Entomol 47: 633-668.

Tooker JF & De Moraes CM (2007) Feeding by Hessian fly [Mayetiola destructor (Say)] larvae does not induce plant indirect defenses. Ecol Entomol 32: 153-161.
